# Structure of cytosine transport protein CodB provides insight into nucleobase‐cation symporter 1 mechanism

**DOI:** 10.15252/embj.2021110527

**Published:** 2022-07-01

**Authors:** Caitlin E Hatton, Deborah H Brotherton, Mahalah Spencer, Alexander D Cameron

**Affiliations:** ^1^ School of Life Sciences University of Warwick Coventry UK; ^2^ Present address: Institute for Nanostructure and Solid State Physics Universität Hamburg, HARBOR Hamburg Germany

**Keywords:** 5‐fluorocytosine, crystal structure, LeuT superfamily, membrane transporter, Membranes & Trafficking, Structural Biology

## Abstract

CodB is a cytosine transporter from the Nucleobase‐Cation‐Symport‐1 (NCS1) transporter family, a member of the widespread LeuT superfamily. Previous experiments with the nosocomial pathogen *Pseudomonas aeruginosa* have shown CodB as also important for the uptake of 5‐fluorocytosine, which has been suggested as a novel drug to combat antimicrobial resistance by suppressing virulence. Here we solve the crystal structure of CodB from *Proteus vulgaris*, at 2.4 Å resolution in complex with cytosine. We show that CodB carries out the sodium‐dependent uptake of cytosine and can bind 5‐fluorocytosine. Comparison of the substrate‐bound structures of CodB and the hydantoin transporter Mhp1, the only other NCS1 family member for which the structure is known, highlight the importance of the hydrogen bonds that the substrates make with the main chain at the breakpoint in the discontinuous helix, TM6. In contrast to other LeuT superfamily members, neither CodB nor Mhp1 makes specific interactions with residues on TM1. Comparison of the structures provides insight into the intricate mechanisms of how these proteins transport substrates across the plasma membrane.

## Graphical abstract

The crystal structure of drug uptake‐linked CodB from *Proteus vulgaris* in a complex with cytosine reveals new mechanistic details.

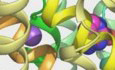

## Introduction

**Figure 1:**
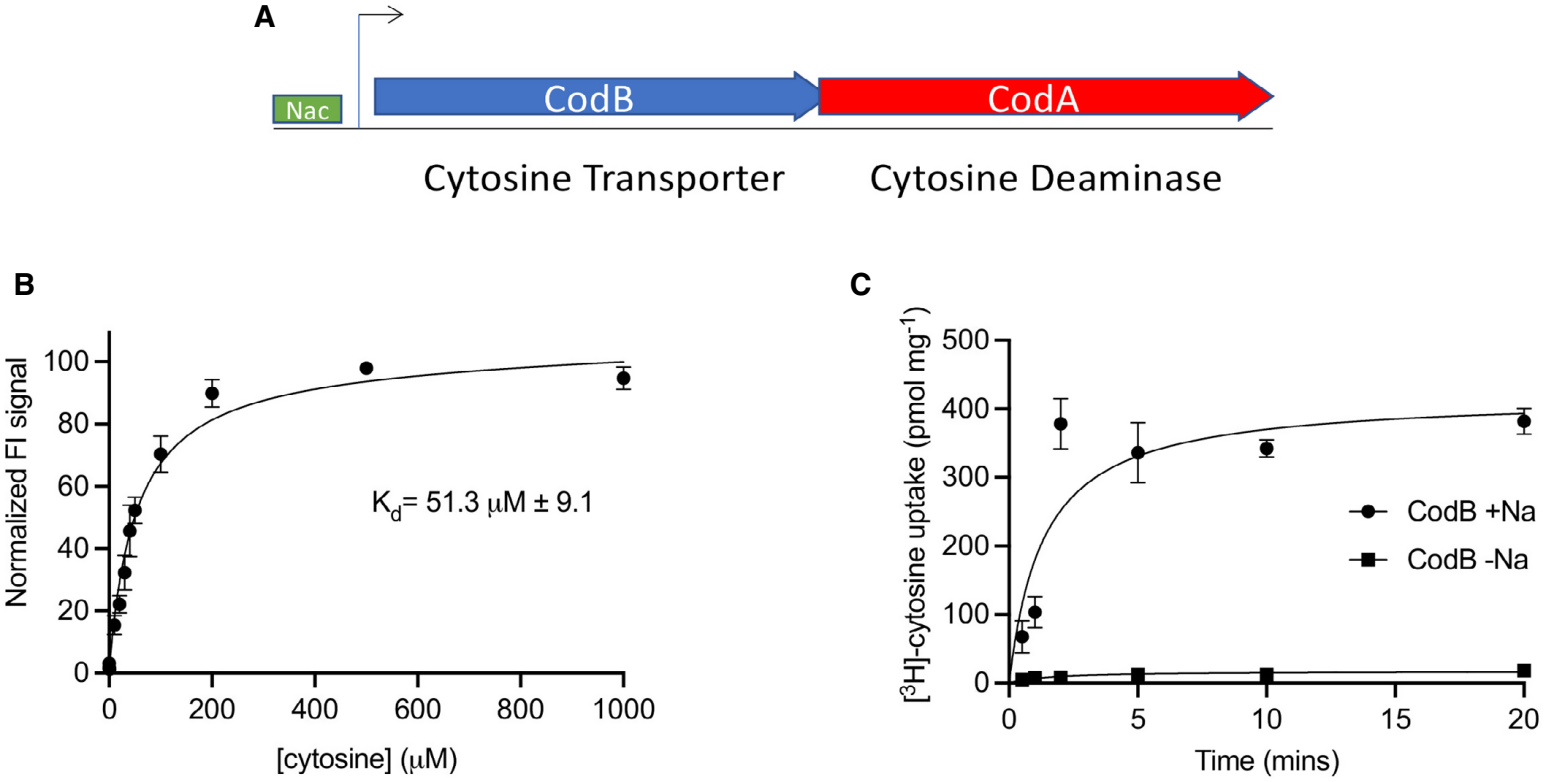
Cytosine binding and transport by CodB AIn *E. coli,* CodB is found in an operon with CodA with overlapping genes. Transcription is regulated by the Nitrogen Assimilation Control protein (NAC) in response to low nitrogen levels (Danielsen *et al*, 1992; Muse *et al*, 2003; Santos‐Zavaleta *et al*, 2019).BBinding affinity of CodB for cytosine as measured using the thermostability assay. Cytosine was titrated into detergent solubilised membranes from cells overexpressing CodB. The K_d_ was estimated to be 51 ± 9 μM. The measurements are the average of 4 independent titrations with error bars of the s.e.m.CTime course of ^3^H‐5‐cytosine uptake by CodB. Experiments were done, either in the presence or absence of an inwardly‐directed sodium ion gradient or with choline chloride. Lemo21 (DE3) cells were used as a background measurement, with Lemo21(DE3) expressing CodB. Values reported are the averaged mean ± s.e.m. from *n* = 3 independent cultures.

The cytosine transporter CodB belongs to the nucleobase cation symporter 1 (NCS1) family of membrane transporters (de Koning & Diallinas, 2000). The NCS1 family is found in bacteria (de Koning & Diallinas, 2000), archaea (Ma *et al*, 2013), fungi (Pantazopoulou & Diallinas, 2007) and plants (Mourad *et al*, 2012; Schein *et al*, 2013; Witz *et al*, 2014). Members of the family are responsible for transporting nucleobases and related molecules into cells, often as components of salvage pathways. In *Escherichia coli*, CodB is found in an operon with CodA, a cytosine deaminase, which converts cytosine to uracil and ammonia, providing an alternative nitrogen source (Fig 1A) (Danielsen *et al*, 1992). In the nosocomial pathogen *Pseudomonas aeruginosa*, CodB has been shown to be important in the effect of 5‐fluorocytosine in the suppression of virulence (Imperi *et al*, 2013). 5‐fluorocytosine is initially taken up by CodB and then converted to toxic 5‐fluorouracil by CodA, which in turn represses the production of bacterial virulence factors resulting in reduced pathogenicity in mouse models of infection (Imperi *et al*, 2013). 5‐fluorocytosine is already used in the clinic as an antimycotic drug (Vermes *et al*, 2000), the toxicity of 5‐fluorouracil being avoided because cytosine deaminases are not found in higher eukaryotes. Drugs that cause a reduction in virulence rather than growth may present a novel means to combat antibiotic resistance as they may not exert the same selective pressure on the organism to develop resistance as traditional antibiotics (Reviewed by Maura *et al*, 2016).

CodB from *E. coli* has a 24% sequence identity to the sodium‐dependent hydantoin transporter, Mhp1 from *Mycobacterium liquefaciens*, the only member of the NCS1 family for which the structure is known (Weyand *et al*, 2008; Shimamura *et al*, 2010; Kazmier *et al*, 2014a; Simmons *et al*, 2014). Determination of the structure of Mhp1 placed the NCS1 family in the amino acid polyamine organocation (APC) transporter or LeuT superfamily (Wong *et al*, 2012). Mhp1, like other members of this superfamily, has a common core built of a pseudosymmetric 5 transmembrane helix, inverted repeat (Abramson & Wright, 2009) with the two repeating units intertwining to give two domains, referred to as the bundle and hash domains in Mhp1. The bundle consists of TMs 1–2 and 6–7 and is characterised by two discontinuous helices (TM1 and TM6). The hash domain is made of TM3‐4 and TM8‐9 (Shimamura *et al*, 2010). Substrates for the respective transporters bind at the interface of the bundle and hash domains near the breakpoints of the two discontinuous helices of the bundle domain. Secondary transporters work by the alternating access mechanism in which the binding site of the protein alternatively faces one side of the membrane or the other (Jardetzky, 1966). The structure of Mhp1 has been solved in the three main states associated with alternating access: outward‐facing with sodium bound (Weyand *et al*, 2008); outward‐facing occluded with sodium and substrate bound (Weyand *et al*, 2008; Simmons *et al*, 2014) and inward‐facing (Shimamura *et al*, 2010). In transitioning between the outward‐facing and inward‐facing states the hash domain moves relative to the bundle domain as an approximate rigid body (Shimamura *et al*, 2010; Kazmier *et al*, 2014a). This mechanism, which is supported by studies using DEER (Kazmier *et al*, 2014a), largely conforms to the rocking bundle model that was first proposed for the leucine transporter LeuT, the founding member of the LeuT superfamily (Forrest *et al*, 2008).

Of the members of the LeuT superfamily that have been solved to date, several, like Mhp1 are sodium coupled. These include LeuT (Yamashita *et al*, 2005), MhsT (Malinauskaite *et al*, 2014), dDAT (Penmatsa *et al*, 2013), SERT (Coleman *et al*, 2016) and GlyT (Shahsavar *et al*, 2021) of the neurotransmitter sodium symporters (NSS) family, vSGLT (Faham *et al*, 2008), SGLT (Han *et al*, 2022; Niu *et al*, 2022) and SiaT (Wahlgren *et al*, 2018) from the solute sodium symporters (SSS) and BetP (Ressl *et al*, 2009) from the betaine/choline/carnitine transporters (BCCTs) family. While the stoichiometry of sodium ions varies amongst the different proteins, the sodium site that is observed in Mhp1 is conserved in all. This site (known as Na2 following its nomenclature in the structure of LeuT) is coordinated by residues at the breakpoint of TM1 of the bundle domain and residues on TM8 of the hash motif. Intuitively, therefore, the conserved sodium site is located at a position that is ideal for stabilising the outward‐facing state of the protein. With respect to these other transporters, Mhp1 is unusual in two respects. Firstly, whereas in the other proteins the respective substrates make critical interactions with the breakpoint of TM1, in Mhp1 there are no direct hydrogen‐bonding interactions between the substrate and TM1, at least as modelled at the limited resolution (3.4 Å) of the substrate‐bound structures. Secondly, whereas studies of the other superfamily members show the position of TM1 varies dependent on the conformational state of the protein (Krishnamurthy & Gouaux, 2012; Perez *et al*, 2012; Kazmier *et al*, 2014b; Coleman *et al*, 2019), in Mhp1 these movements are much more subtle (Shimamura *et al*, 2010; Kazmier *et al*, 2014a; Simmons *et al*, 2014).

CodB transports cytosine, a much smaller compound than the bulky substituted hydantoins transported by Mhp1. To understand how cytosine and 5‐fluorocytosine bind in the substrate‐binding site we solve the crystal structure of the protein in complex with cytosine and a sodium ion at 2.4 Å resolution. Combining this data with transport assays and site‐directed mutagenesis provides insight into molecular recognition and transport in CodB and the NCS1 family and indeed the APC superfamily in general.

## Results

### CodB is a sodium‐dependent cytosine transporter

**Figure EV1:**
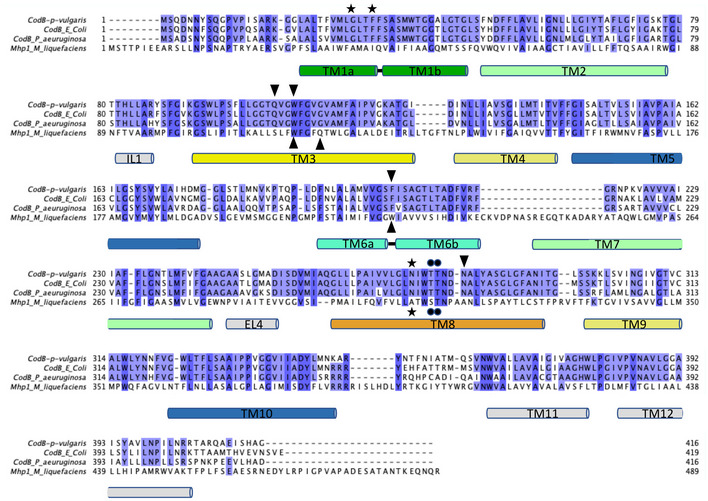
Sequence alignment of CodB and Mhp1 Alignment of sequences of CodB from *P. vulgaris*, *E. coli* and *P. aeruginosa* with Mhp1 from *M. liquefaciens* shaded according to sequence conservation. The sequences were aligned with Muscle (Edgar, 2004) with manual adjustments based on the respective structures. The secondary structure is shown for CodB. Residues interacting with the substrate are denoted by triangles (▼ for CodB and ▲ for Mhp1), residues interacting with the sodium ion through the carbonyl oxygen are shown as ★ and those interacting through the side chain are denoted by ●.

CodB from the opportunistic pathogen *Proteus vulgaris* (CodB_PV_) was identified as suitable for structural studies using fluorescent‐based screening methods (Drew *et al*, 2006; Sonoda *et al*, 2011). CodB_PV_ has 84% sequence identity with CodB from *Escherichia coli* and 74% identity with that from *P. aeruginosa* (Fig EV1). As for *E. coli*, in both *P. vulgaris* and *P. aeruginosa*, the gene encoding CodB is found in an operon with CodA. In a stabilisation assay (Nji *et al*, 2018) cytosine was observed to stabilise the detergent solubilised protein (Fig 1B). Using stabilisation as a surrogate for binding, the affinity was measured to be ~ 50 μM. Although there are no reports of sodium‐dependency in CodB, given that the residues involved in sodium ion coordination are conserved between CodB and Mhp1 (Fig EV1) we suspected that, like Mhp1, the transporter would be sodium coupled. Sodium‐dependent uptake of cytosine was confirmed using an in‐cell transport assay by following the uptake of ^3^H‐cytosine (Fig 1C; Appendix Fig S1).

### Overall structure and conformation of CodB

**Figure 2:**
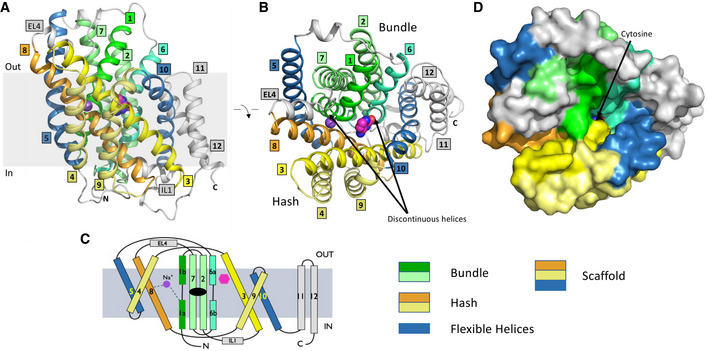
Structure of CodB ARibbon diagram of CodB in the plane of the membrane. The bundle motif is depicted in different shades of green with TM1 in green, TM6 in sea green and TMs 2 and 7 in light green. The hash motif is shown with TM3 in yellow, TM8 in orange and TMs 4 and 9 in light yellow. The flexible helices TM5 and TM10 have been coloured blue. In other LeuT superfamily members, the combination of the hash motif and the flexible helices is often referred to as the scaffold domain (Forrest *et al*, 2008). TM11 and TM12 are coloured grey. EL4 is the extracellular loop linking TMs 7 and 8 and IL1 is the intracellular loop between TMs 2 and 3. The carbon atoms of the cytosine are coloured magenta. The sodium ion is depicted as a purple sphere. The approximate position of the membrane is denoted by the shaded box.BAs (A) but looking from the extracellular side of the membrane.CTopology diagram coloured as in a.DSurface representation in the same colouring as (A and B) with the same view as (B). The cytosine can be observed at the bottom of an open cavity.

**Figure 3:**
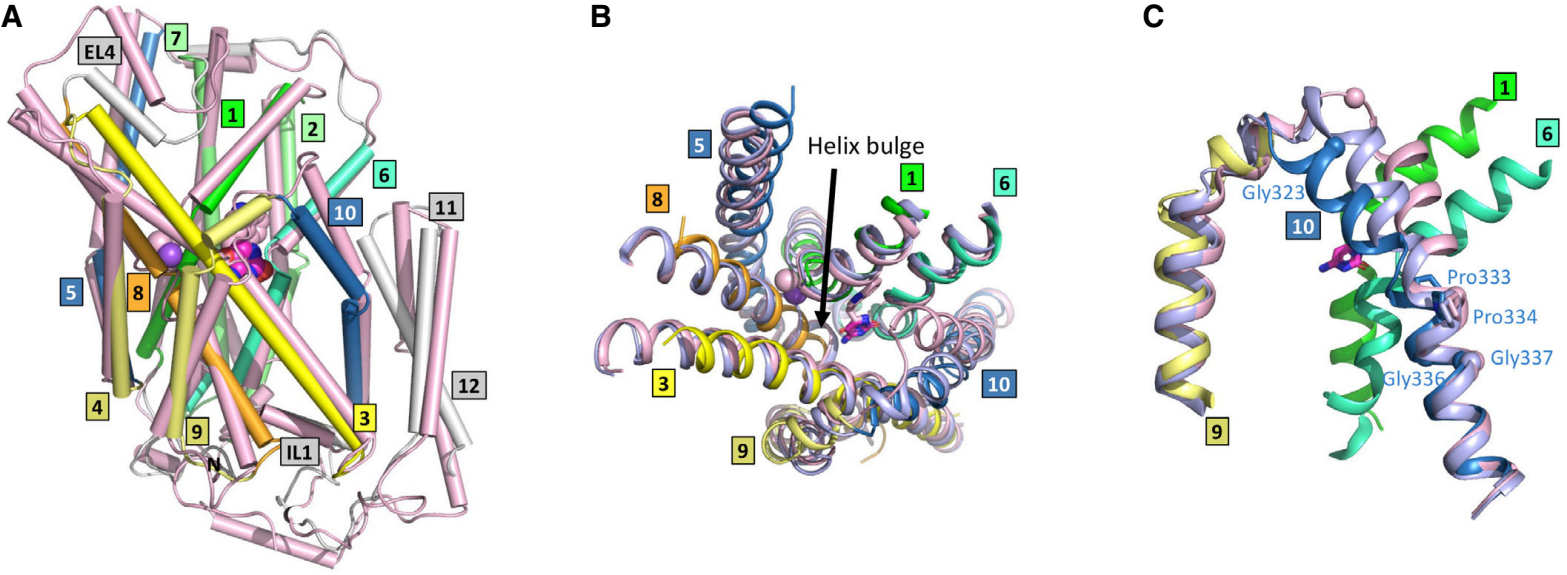
Comparison of the structures of CodB and Mhp1 AOverlay of the complete structures with CodB coloured as in Fig 2 and the occluded form of Mhp1 in pink. Comparing CodB with the outward‐occluded form of Mhp1 (4D1A), CodB aligned with a RMSD of 2.0 Å for 344 residues out of a possible 416. These differences are distributed throughout the protein. When the 4‐helix bundle and hash domain were extracted and aligned the respective RMSDs were 2.1 Å for 123 out of 152 residues for the bundle domain and 1.7 Å for 99 out of 119 residues for the hash domain.BView of the sodium and cytosine/hydantoin‐binding sites from the extracellular side.CComparison of TM10 in the structures of CodB (blue), the outward‐open form of Mhp1 (2JLN lilac) and the occluded form of Mhp1 (4D1A pink). TMs 9 and 10 are shown for all structures. For CodB TMs 1 and 6 are also shown to help orientation. Glycines are denoted by spheres at the C_α_ atoms and prolines as sticks. The glycine and proline residues on TM10 of CodB are labelled.

**Figure EV2:**
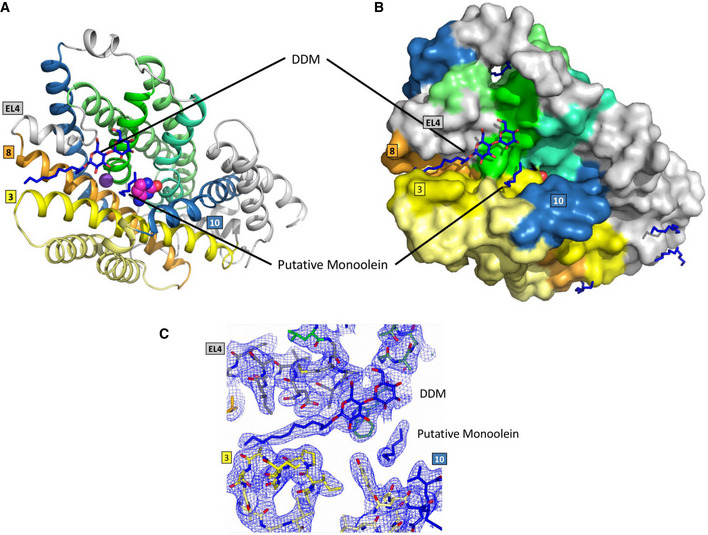
Detergent and lipid binding in CodB As Fig 2B but showing positions of monoolein and DDM that have been putatively modelled in the binding site. Lipids are shown with blue carbon atoms. A–C(A) cartoon representation, (B) surface representation. (C) Electron density for lipid‐like molecules in the cavity. The 2mFo‐DFc map is based on phases from the refined structure and contoured at 0.75σ.

CodB was purified and crystallised in the presence of cytosine using the lipidic cubic phase (LCP) method (Caffrey & Cherezov, 2009) and the structure was determined and refined at 2.4 Å to an Rfactor of 20.1% and a corresponding Rfree of 24.6% (Table 1) with excellent density (Appendix Fig S2). The addition of cytosine during purification was observed to reduce protein loss, consistent with stabilisation of the protein. CodB crystallises as a monomer with two molecules in the asymmetric unit orientated oppositely with respect to the membrane plane. Both molecules adopt an outward‐open conformation (Fig 2A and B and D, and EV2) with cytosine bound in a solvent accessible polar pocket and density consistent with a Na^+^ ion observed in the conserved Na2 site. The overall 12‐TM helix topology is very similar to Mhp1: TM1‐TM5 are related to TM6‐TM10 by a pseudo 2‐fold axis and intertwine to form the bundle and hash motifs (Fig 2C). Transmembrane helices TM11‐TM12 abut the hash motif. Between EL4 and the tip of TM3, TM10 and TM1 there is non‐protein density, which we have tentatively modelled as DDM and monoolein respectively (Fig EV2). Overall, the root mean square deviation (RMSD) between CodB and the occluded form of Mhp1 is 2 Å for 344 Cα atoms out of a possible 416 (Fig 3A–C). TM8 forms a more regular helix than seen in Mhp1 where there is a single residue insertion into the helix next to the substrate‐binding site (Figs 3B and EV1), but most of the substantial differences are in the loop regions where the sequence of CodB is generally shorter than Mhp1 (Figs 3A and EV1). The helix that forms part of EL4, which is critical in sealing the extracellular cavity on the transition to the inward‐facing form (Shimamura *et al*, 2010; Kazmier *et al*, 2014a) in Mhp1 is set more deeply into the cavity (Fig 3A). In Mhp1, upon substrate binding, TM10 bends towards the substrate. In CodB this transmembrane helix is in a position more reminiscent of the non‐substrate bound outward‐open form of Mhp1 rather than the substrate occluded form (Fig 3B and C).

**Table 1:** Data processing and refinement statistics.

	Low resolution	High resolution
Wavelength (Å)	0.9686	0.9686
Resolution range	52.4–3.6 (3.7–3.6)	59–2.4 (2.49–2.4)^a^
Space group	C 2 2 2_1_	C 2 2 2_1_
Unit cell: a, b, c (Å)	108.5, 209.5, 102.8	108.2, 209.0, 102.4
Total reflections	72,321 (7,329)	160,886 (13,618)
Unique reflections	13,943 (1,337)	45,528 (4,436)
Multiplicity	5.2 (5.3)	3.5 (3.1)
Completeness (%)	95.6 (97.0)	99.41 (98.64)
Mean I/sigma(I)	3.5 (0.9)	7.71 (0.94)
Wilson B‐factor	34.5	42
R‐merge	0.5 (1.2)	0.1829 (1.085)
R‐meas	0.6 (1.4)	0.2152 (1.308)
R‐pim	0.3 (0.6)	0.1108 (0.7114)
CC1/2	0.7 (0.5)	0.908 (0.424)
CC^a^	0.9 (0.8)	0.976 (0.772)
Reflections used in refinement	13,353 (1,337)	45,487 (4,428)
Reflections used for R‐free	1,335 (133)	2,208 (215)
R‐work	0.3 (0.4)	0.2010 (0.2640)
R‐free	0.3 (0.4)	0.2461 (0.3339)
CC (work)	0.6 (0.4)	0.898 (0.760)
CC (free)	0.7 (0.3)	0.921 (0.691)
Number of non‐hydrogen atoms	5,711	6,139
Macromolecules	5,695	5,899
Ligands	16	175
Solvent	0	65
Protein residues	793	803
RMS (bonds)	0.01	0.008
RMS (angles)	1.4	0.88
Ramachandran favoured (%)	95.7	98.75
Ramachandran allowed (%)	3.7	1.13
Ramachandran outliers (%)	0.6	0.13
Rotamer outliers (%)	7.7	1.80
Clashscore	23.2	8.74
Average B‐factor	19.98	52.52
Macromolecules	20.04	52.00
Ligands	0.50	71.15
Solvent		49.49

a Numbers in parenthesis refer to the highest shell.

### Cytosine‐binding site

**Figure 4:**
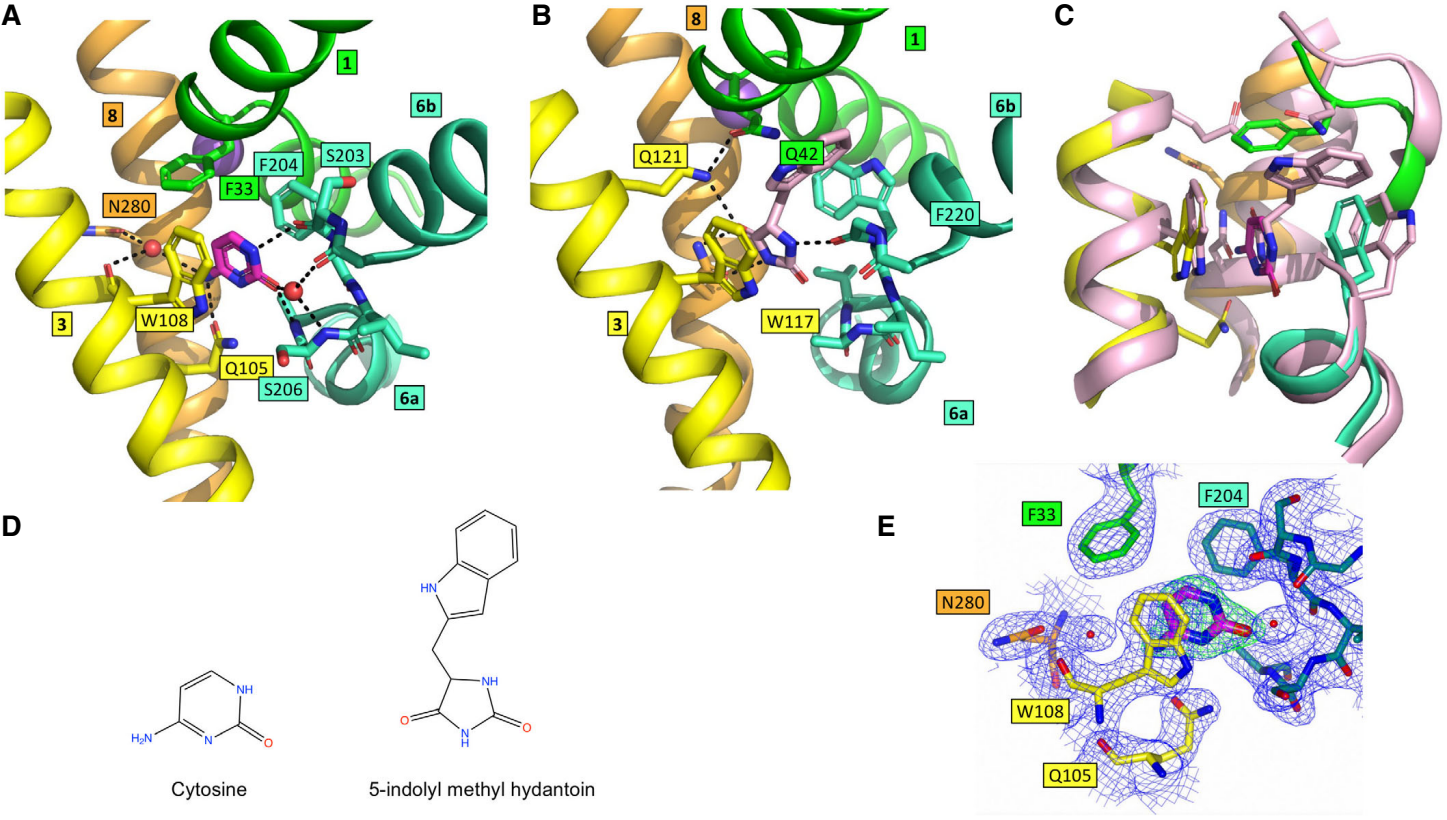
Comparison of the substrate‐binding sites in CodB and Mhp1 AView of the binding site of CodB coloured as in Fig 1. Water molecules are denoted as red spheres and potential hydrogen bonds are shown as dashed lines.BMhp1 in the same view as (A) and coloured as for CodB. The indolylmethyl‐hydantoin is shown with pink carbon atoms.COverlay of CodB and Mhp1 with CodB coloured as in (A) and Mhp1 in pink.DChemical structures of the respective substrates.EElectron density for the cytosine. The 2mFo‐DFc map in blue is contoured at 1σ and the mFo‐DFc map, calculated before the addition of the cytosine, in green at 3σ.

The cytosine substrate is found at the interface of the hash‐motif and the 4‐helix bundle, sandwiched between two aromatic residues, Trp108 of TM3 of the hash domain and Phe204 of TM6 of the bundle domain (Fig 4A and E) in a face‐to‐face pi stacking arrangement. The cytosine forms two direct hydrogen bonds to the main chain at either side of the breakpoint of TM6 of the bundle domain: to the carbonyl oxygen of Ser203 of TM6a and to the main chain nitrogen atom of Ala207 of TM6b (Fig 4A). In addition, a water molecule bridges the cytosine to the carbonyl oxygens of Gly202 (TM6a) and Ser206 (TM6b). In terms of interactions with the hash motif, as well as stacking with Trp108, there is a hydrogen bond from the cytosine to the amino oxygen of Gln105 of TM3 and a potential water‐mediated hydrogen bond to Asn280 of TM8.

When the substrate‐binding site in CodB is compared to Mhp1 and other members of the NCS1 family, the relative importance of the interactions that the base makes with the protein can be inferred. The two aromatic residues, which sandwich the cytosine in CodB, are conserved throughout the NCS1 family; whereas the equivalent of Trp108 is predominantly a tryptophan, the equivalent of Phe204 can be either a phenylalanine as seen in CodB or a tryptophan as in Mhp1. The remaining residues that interact with the cytosine in CodB are much less conserved throughout the family. In Mhp1 the major hydrogen‐bonding interactions between the substrate and the residues from the hash motif are with Asn318 from TM8 and Gln121 from TM3 rather than the equivalent of Gln105. Both substrates, from CodB and Mhp1, however, are within hydrogen‐bonding distance of TM6. Whereas in CodB the cytosine interacts with the main chain atoms of both TM6a and TM6b on either side of the helix break, in Mhp1 only TM6a is within hydrogen‐bonding distance of the hydantoin (Fig 4B). The equivalent interaction between the substrate and TM6b to that seen in CodB is ~ 3.8 Å, slightly too long for a hydrogen bond, although it is possible that this also reflects the resolution of the Mhp1 structure (3.4 Å) and with minor adjustments of the positioning of the base and/or the main chain atoms could bring the two atoms to a position more consistent with a hydrogen bond. What is remarkable is that when the structure of CodB is superposed on that of Mhp1 based on their respective Cα atoms, the cytosine of CodB and the hydantoin moiety of the Mhp1 substrate overlap almost exactly (Fig 4C). This is surprising given that firstly, the substrates of the two proteins are different (Fig 4D), and secondly, there is limited conservation within the binding sites. The fact that the cytosine and the hydantoin moiety of the respective substrates overlap so well demonstrates the importance of the interactions with TM6 as well as the aromatic residues. It is noteworthy that after superposing the two proteins as above, the phenyl ring of CodB overlaps the 6‐membered ring of the tryptophan in Mhp1 (Fig 4C).

Neither CodB nor Mhp1 has specific interactions involving the respective substrates and TM1. The only interaction that the cytosine makes with TM1 is a potential edge‐to‐face pi‐stacking arrangement with Phe33. The equivalent residue in Mhp1 is Gln42, which is not involved in hydrogen bonding the substrate, but is within hydrogen‐bonding distance of Gln121.

### Sodium‐binding site

**Figure 5:**
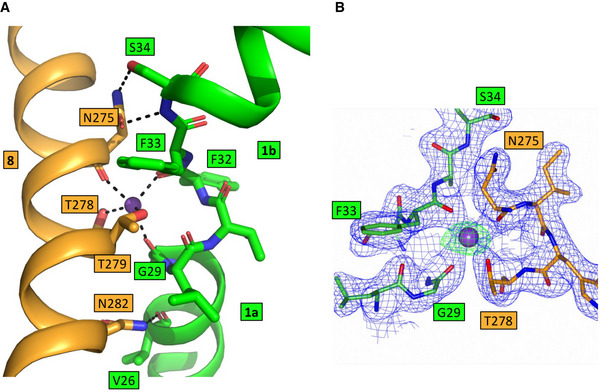
Sodium‐binding site in CodB AView showing the interactions between TM1 and TM8 centred on the sodium ion. The sodium ion makes interactions with residues on TM1 and TM8 (black dashed lines). Asn275 and Asn282 are also within hydrogen‐bonding distance of residues on TM1.BElectron density associated with the sodium ion. The 2mFo‐DFc map in blue is contoured at 1σ and the mFo‐DFc map, calculated before the addition of the sodium ion is in green at 5σ.

**Figure 6:**
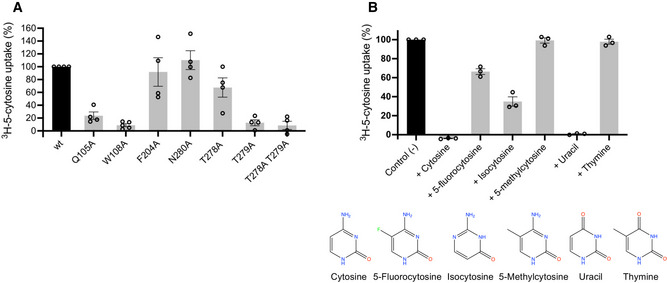
Functional characterisation of CodB AUptake of ^3^H‐5‐cytosine by CodB mutants relative to the wild‐type protein. Uptake of ^3^H‐5‐cytosine was measured after 1 min. Uptake for the wild‐type protein was set at 100%, and the mutants are shown as a percentage of this with error bars as s.e.m. of at least 4 experiments, each from a different culture.BInhibition of ^3^H‐5‐cytosine uptake in the presence of 0.1 mM of each respective inhibitor. Uptake of ^3^H‐5‐cytosine was measured after 1 min with 0.1 mM inhibitor. Control (−) is uptake of ^3^H‐5‐cytosine with no inhibitor, normalised to 100%, results are visualised as % of control (−) with error bars as s.e.m. of triplicate experiments, each from a different culture. The chemical structures of the ligands are shown below the graph.

**Figure EV3:**
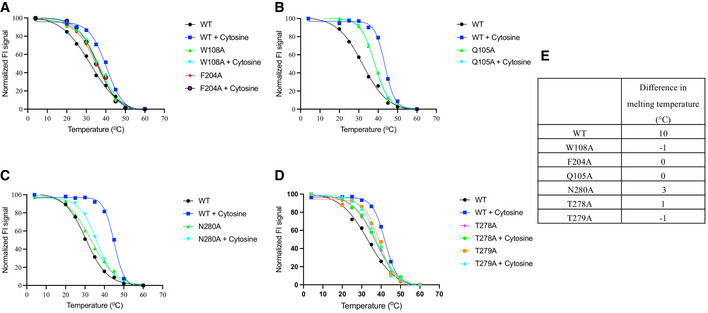
Characterisation of CodB mutants using the thermostability assay A–DMelting curves for wild‐type and mutant proteins with and without cytosine bound using the GFP‐TS assay. Values plotted are the averages from *n* = 2 independent titrations, each with two technical repeats.ETable summarising the data from the graphs in (A–D) showing differences in melting temperatures between each protein with and without cytosine bound.

Electron density consistent with a sodium ion is visible at the Na2 site that is conserved amongst the Na^+^‐coupled LeuT transporters (Fig 5B). The sodium ion is coordinated by the main chain carbonyl oxygens of Gly29 and Phe32 at the breakpoint of TM1 of the bundle domain and the main chain carbonyl oxygen of Asn275 and the hydroxyl oxygens of Thr278 and Thr279 from TM8 of the hash domain in a square pyramidal arrangement (Fig 5A and B). In contrast to the wild‐type protein, protein with either Thr278 or Thr279 substituted with alanine was not stabilised by the addition of cytosine (Fig EV3D), consistent with sodium binding at this position being necessary for cytosine binding. Mutation of Thr279 to alanine also caused a marked reduction in the transport of ^3^H‐cytosine, although under the conditions of the transport assay the same mutation of Thr278 had little effect (Fig 6A). In an unusual interaction that is not seen in other Na^+^‐coupled LeuT members, the side chain of Asn275 is also within hydrogen‐bond distance of the side chain hydroxyl and the amide nitrogen of Ser34 at the C‐terminus of TM1b, providing a further link between the hash and bundle domains when sodium binds (Fig 5A). More typically hydrophobic residues are found at this position. Interestingly, Asn282, also on TM8 and positioned just below the sodium ion, in the view shown in Fig 5A, also forms hydrogen‐bonding interactions to Val26 of TM1a bridging these two helices (Fig 5A).

### Molecular recognition in CodB

**Figure EV4:**
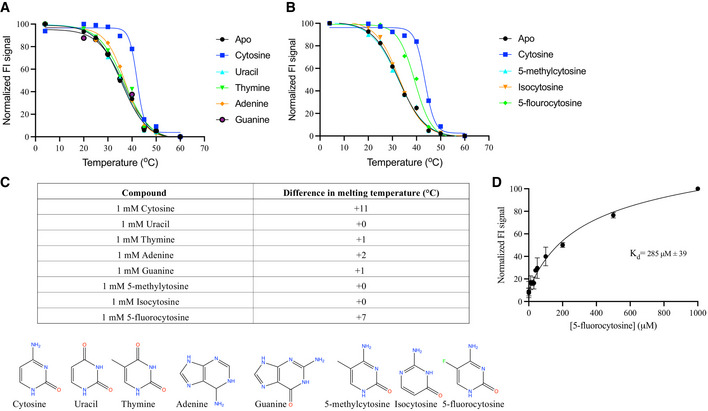
Characterisation of nucleobase binding to CodB using the thermostability assay A, BMelting curves for the wild‐type CodB in the presence and absence of selected nucleobases using the GFP‐TS assay. Values plotted are the averages from *n* = 2 independent titrations, each with two technical repeats.CTable summarising the graphs from (A, B) showing differences in melting temperatures upon addition of the specified nucleobase.DBinding affinity of CodB for 5‐fluorocytosine as measured using the thermostability assay. The K_d_ was estimated to be 285 ± 39 μM. The measurements are the average of 3 independent titrations with error bars of the s.e.m.

The three key residues that interact with the cytosine through their side chains are Gln105, Trp108 and Phe204. While mutation of any of these residues to alanine caused an apparent reduction in binding of the cytosine, as monitored through the stabilisation assay (Fig EV3A and B and E), only mutations of Gln105 or Trp108 caused a dramatic reduction in the transport of ^3^H‐cytosine (Fig 6A). Mutation of Asn280, which forms a water‐mediated hydrogen bond had no effect on transport, though it did appear to affect binding (Figs 6A and EV3C and E). To investigate the specificity of CodB for cytosine a selection of nucleobases and related compounds were tested for their effect on stabilising the protein or in inhibiting transport of ^3^H‐cytosine. Of the bases investigated, cytosine was the most effective both at stabilising the protein and inhibiting transport (Figs 6B and EV4A–C). Consistent with its effect on *P. aeruginosa* (Imperi *et al*, 2013) 5‐fluorocytosine also showed some inhibition of cytosine uptake (Fig 6B) with a K_D_ estimated from the stability assay of 285 μM (Fig EV4D). It would seem likely that this would bind in a similar mode to cytosine with the fluorine interacting with Phe33. Methylcytosine, where the fluorine is replaced with a much larger methyl group on the other hand does not bind, presumably because the methyl group is likely to clash with Phe33. Although, both uracil and isocytosine inhibited the uptake of ^3^H‐cytosine under the conditions of the uptake assay, they had little effect in stabilising the protein in the stabilisation assay (Fig EV4A and B). Modelling of the uracil into the pocket, based on the cytosine‐binding mode, suggests that the uracil may be able to bind if the side chain of Gln105 were to flip and this may also occur with isocytosine. No binding was observed for the purine bases.

### Further interactions between bundle and hash domains

**Figure EV5:**
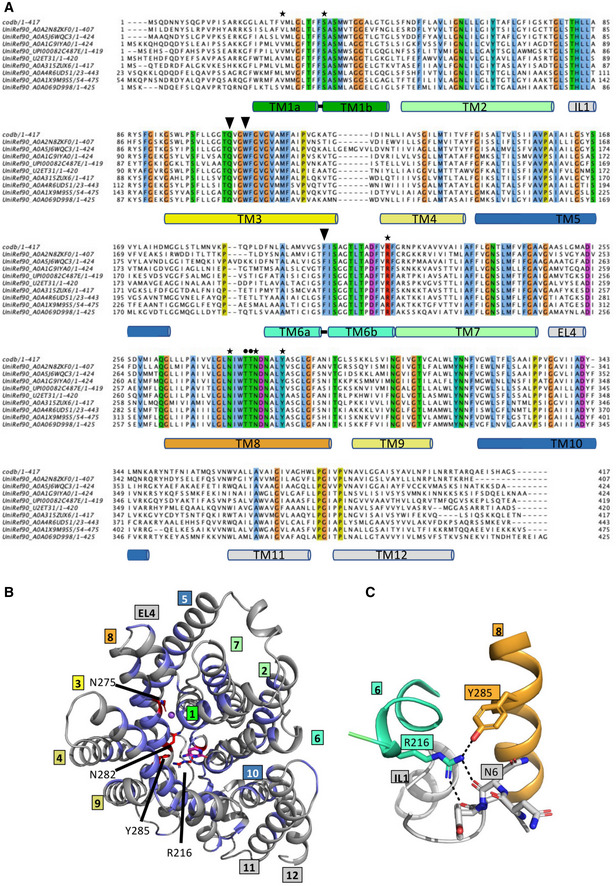
Sequence conservation in CodB AA BLAST search was carried out against UniProt clusters (UniRef90). The most diverse sequences from the 200 sequences deriving from that search (see methods) are shown. Residues that are identical in all 10 sequences are highlighted colored according to ClustalX. Residues interacting with the ligand are shown by a solid triangle (▼), those interacting with the sodium ion through their side chains are shown as a ● and those involved in hydrogen bonds between the helices of the bundle and hash domain respectively are denoted with a ✶. As more diverse sequences are added R216 and Y285 remain constant while there is more divergence for other residues.BConservation plotted on the structure of CodB. Residues that are identical in (A) have been coloured blue with N275, N282, R216 and Y285 depicted as red sticks.CInteraction between Arg216 and Tyr285 coloured as in Fig 2. Hydrogen bonds are shown as dashed lines.

By investigating the pattern of conservation amongst CodB homologues we discovered that Arg216 on TM6b of the bundle domain and Tyr285 of TM8 of the hash domain are two of the most conserved residues (Fig EV5A and B). Remarkably, these residues are within hydrogen‐bonding distance of one another at the cytoplasmic side of the protein (Fig EV5C). The high conservation suggests this interaction may be important for function. The same interaction is not found in Mhp1, however, in Mhp1 the arginine is replaced with a lysine (Lys232) and though the tyrosine is not conserved, the hydroxyl oxygen of Tyr324 one helix turn down is positioned such that a similar interaction would be possible (Appendix Fig S3A).

## Discussion

The structure of CodB we have elucidated here shows how the cytosine substrate makes specific hydrogen‐bonding interactions with the exposed main chain atoms at the breakpoint of TM6. Comparison with Mhp1 clearly shows that this is the common recognition site between these distantly related members of the NCS1 family. In other sodium‐coupled members of the LeuT superfamily (Yamashita *et al*, 2005; Faham *et al*, 2008; Perez *et al*, 2012; Malinauskaite *et al*, 2014; Penmatsa *et al*, 2015; Coleman *et al*, 2016; Wahlgren *et al*, 2018) interactions with TM1 appear to be more important in anchoring the substrate than those of TM6 and those with TM6 to be more modulatory. In the NSS protein MhsT, for instance, the flexibility of the residues at the breakpoint of TM6 allows the accommodation of different amino acids (Focht *et al*, 2021). The sugar substrate in vSGLT is possibly the exception in not interacting with the main chain of TM1 (Faham *et al*, 2008), but this is an inward‐facing structure where the binding site is not fully formed. Interactions with the main chain of TM1 are seen in the outward‐facing sialic acid transporter, SiaT from the same family (Wahlgren *et al*, 2018). In CodB the only interaction between the substrate and TM1 is a stacking interaction with Phe33. Mutagenesis of the equivalent residue in Mhp1 led to the conclusion that the only function of this residue would be to shape the pocket (Simmons *et al*, 2014) and the position of Phe33 in CodB would support this conclusion.

Clearly, the pi‐stacking arrangement of the nucleobase between the two aromatic residues is also important. Interestingly, the mutation of Phe204 of TM6 of the bundle domain to Ala was much less drastic compared to the similar mutation of the hash motif residue Trp108. A similar observation was made with the equivalent mutation in Mhp1 (Simmons *et al*, 2014). It seems likely, therefore, that the prime binding site on the bundle domain involves the main chain atoms of TM6 with Phe204 contributing to the overall shape of the pocket, though without a crystal structure of the mutant protein, we cannot rule out structural changes caused by the mutation of Phe204 to counteract the loss of the aromatic side chain. Subtle changes caused by the interaction with the residues on the hash domain may then be important in allowing transport to occur. 5‐Fluorocytosine could easily be accommodated with the same binding mode.

For alternating access to occur there are several changes that have been shown to take place in Mhp1. Following substrate binding, TM10 folds into the binding site, rotating around a conserved proline (Weyand *et al*, 2008). In our structure TM10 adopts a more open conformation. Although TM10 in CodB is one residue shorter than in Mhp1, given that the temperature factors are high for TM10 (Appendix Fig S4) and the helix retains the proline on TM10 around which TM10 swivels (Fig 3C) it seems likely that the lipid‐like molecules that we observe in the density are preventing the conformation adopted in the substrate‐bound form of Mhp1 rather than a substantial difference in mechanism. Molecular dynamics (Shimamura *et al*, 2010) and DEER (Kazmier *et al*, 2014b) both suggest this helix is very mobile in the outward‐facing structure of Mhp1.

The second major conformational change in the transport cycle involves a rotation of the hash domain relative to the bundle domain (Shimamura *et al*, 2010). It can be speculated that this transition is triggered by the movement of TM10 towards the substrate, which will necessarily affect TM9 of the hash domain. The rotation in Mhp1 is around an axis that is approximately coincidental with TM3 so that the movement of TM8 as the protein transitions from outward to inward facing is much greater than that of TM3. Intuitively, it would be thought that the sodium ion, which spans TM1 and TM8 in the outward‐facing structure is likely to be important in shifting the equilibrium towards the outward‐facing state. For LeuT, studies using DEER are consistent with this (Kazmier *et al*, 2014b). In contrast, in studies of Mhp1 using DEER and mass spectrometry, the presence of the substrate as well as sodium ions was required to drive the conformational change from inward to outward‐facing states in detergent solution (Kazmier *et al*, 2014a; Calabrese *et al*, 2017). Given that the crystal structure of Mhp1 in the presence of sodium ions but without substrate is outward‐facing it seems likely that subtle changes in the energetics of the system, such as the lipid environment or membrane potential are likely to influence the conformational state of the protein. In Mhp1, Asn318 on TM8 makes an important bidentate hydrogen‐bonding interaction with the substrate so this is likely to influence the conformational change. In CodB, on the other hand, there is only a water‐mediated interaction between TM8 and the cytosine. Instead, there are direct hydrogen‐bonding interactions between TM8 and TM1, one involving Asn275, which is also a ligand to the sodium ion and the other from Asn282 which is just below the sodium ion. These residues may affect the activity of the protein, albeit subtly, by making it energetically more favourable to adopt the outward‐facing state in the presence of sodium ions. It is noteworthy that in the sialic acid transporter, SiaT, the equivalent residue to Asn282 is involved in a second sodium‐binding site, which appears to modulate activity (Wahlgren *et al*, 2018). In general, the hydrogen‐bonding arrangement between residues of the hash domain and residues of the bundle domain in CodB differ widely from those in Mhp1. In Mhp1 there are no residues from TM8 that are involved in direct hydrogen‐bonding interactions with the bundle domain, but instead two residues from TM3, (Gln121 and Lys110, Appendix Fig S3B).

Given the conservation of Tyr285 and Arg216 amongst putative CodB homologues from different organisms, the interaction between them appears to be important. This interaction is reminiscent of that between Tyr268 and Gln361 of LeuT (Yamashita *et al*, 2005), which is conserved in the NSS family. The mutation of Tyr268 in LeuT favours the inward‐open structure (Krishnamurthy & Gouaux, 2012; Kazmier *et al*, 2014b). CodB also resembles LeuT in that Arg216 also interacts with the N‐terminus (Fig EV5C). Though there is no conservation in the residues involved, the interaction between Arg5 at the N‐terminus of LeuT and Asp369 within the scaffold domain is important in the mechanism of LeuT and NSS transporters (Kniazeff *et al*, 2008; Krishnamurthy & Gouaux, 2012). It has been shown for other NCS1 members that the N‐terminus affects the mechanism and specificity of the transporters (Papadaki *et al*, 2019). It therefore seems that while each of the proteins has important interactions linking the two domains, the exact mode widely varies amongst them.

**Figure 7:**
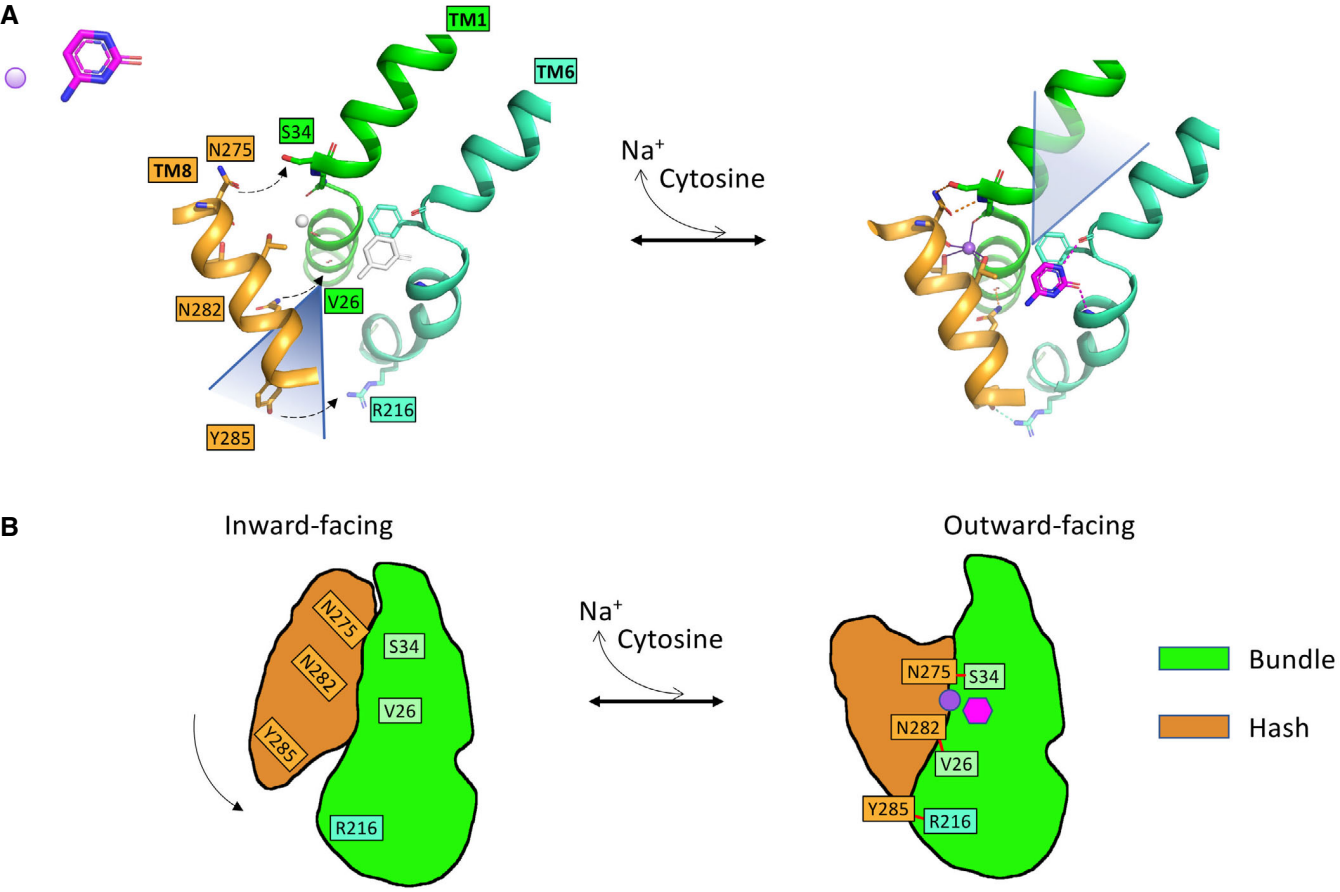
Mechanism of transition from inward to outward facing conformations in CodB AThe putative movement of TM8 relative to the bundle as the protein transitions from the inward‐facing state (left) to the outward‐facing state (right). The position of TM8 in the inward‐facing state has been modelled on the equivalent helix of Mhp1 in the inward‐state (PDB code 2X79). In transitioning between the two states TM8 rotates around an axis approximately coincidental with TM3 bringing it much closer to TM1 so that the hydrogen‐bonding interactions seen in the outward‐facing state (dashed lines) can form and the sodium ion can bind. These distances are too large for hydrogen bonds in the modelled inward‐facing state. Cytosine will bind guided by residues on TM6 as well as TM3 (not shown) enabling conformational changes that will result in the transition back to the inward‐facing state. The inward and outward facing clefts, which lie to the back of TM8 in the inward‐facing state and to the front of TM1 in the outward‐facing state are denoted by triangles behind and in front of the cartoons, respectively.BA schematic showing the interactions between TM1 and TM8 acting like a zipper on the protein.

In conclusion, the high‐resolution structure of CodB with cytosine in combination with site‐directed mutagenesis has enabled us to understand substrate binding in CodB and see that 5‐fluorocytosine could easily be accommodated in the binding site. Given the complete conservation of the residues in the cytosine‐binding site between CodB from *P. vulgaris* and from *P. aeruginosa* this is directly translatable to the pathogenic organism. Any modifications of 5‐fluorocytosine, to make it a more potent drug, could therefore take into account whether the molecule would be taken up by CodB. The structure also illustrates the importance of the interaction between the substrate and TM6 in the NCS1 family. The structural analysis highlights how the interaction with the sodium ion and substrate are separated, with the sodium ion binding to TM1 and the substrate primarily interacting with TM6 (Fig 7A) unlike the arrangement in other characterised members of the superfamily. Whether this can be correlated with the larger movements of TM1 seen in other members of the superfamily during the transport cycle remains to be seen. Both mechanisms are compatible with the movement of the bundle relative to the hash motif that is observed in the superfamily. It seems likely that the three hydrogen‐bonding interactions between residues of TM1 and TM8 discussed above, will also influence the mechanism. Presumably these interactions will stabilise the outward‐facing state of the protein in readiness for the cytosine to bind (Fig 7A and B). The structural analysis provides further insight into how a common mechanism of sodium‐coupled symport in this superfamily is modulated by structurally similar proteins in diverse ways.

## Materials and Methods

### Expression and protein purification

The gene encoding for the cytosine permease CodB from *P. vulgaris*, codon optimised for expression in *E. coli* was purchased as a gBlock (Integrated DNA Technologies). This was inserted into a modified version of the expression vector, pWaldo GFPd (Drew *et al*, 2006) in which the TEV protease site had been altered to a site for recognition by 3C protease (see Appendix). Site‐directed mutations were introduced by PCR (Quikchange, Agilent Technologies; Appendix Table S1). CodB‐GFP fusions were expressed in *E. coli* Lemo21 (DE3) cells following the MemStar procedure (Lee *et al*, 2014). Briefly, cultures were grown at 37°C, 200 rpm, in PASM‐5052 media supplemented with 0.1 mM rhamnose. When cultures reached OD_600_ = 0.5, the temperature was dropped to 25°C and 0.4 mM IPTG was added for protein induction overnight.

Cell pellets were harvested by centrifugation and resuspended in PBS (137 mM NaCl, 2.7 mM KCl, 10 mM Na_2_HPO_4_, 1.8 mM KH_2_PO_4_) with 1 mM MgCl_2_, DNaseI, and 0.5 mM 4‐benzenesulfonyl fluoride hydrochloride (AEBSF) and disrupted by passing three times through a cell disruptor at 25 kPsi. Cell lysate was centrifuged at 24,000 *g* at 4°C for 12 min to remove insoluble cell debris, and the supernatant was subjected to ultracentrifugation at 200,000 *g*, 4°C for 45 min. Membrane pellets were resuspended in PBS, 15 ml per 1 l of culture, snap frozen in liquid nitrogen, and then stored at −80°C.

For crystallisation membranes from 3 l of culture were solubilised in 1x PBS, 150 mM NaCl, 1% DDM for 2 h at 4°C and ultracentrifuged for 45 min, 4°C, 200,000 *g* to remove insoluble material. Imidazole was added to 20 mM, and the membrane suspension was mixed with 1 ml of Ni‐NTA Superflow resin (Qiagen) per 1 mg of GFP–His8 and incubated for 3 h at 4°C. Slurry was decanted into a glass Econo‐Column (Bio‐Rad) and washed with 5 Column Volumes (CV) of 1x PBS, 150 mM NaCl, 20 mM imidazole, 0.1% DDM, then 5 CV of 20 mM TRIS pH 7.5, 150 mM NaCl, 30 mM imidazole, 0.03% DDM, 1 mM cytosine. Protein was left on the column overnight with a 1:1 stoichiometry of 3C protease at 4°C. Cleaved protein was eluted into fractions corresponding to 1CV and passed over a 5 ml HisTrap equilibrated with 20 mM TRIS pH 7.5, 150 mM NaCl, 30 mM imidazole, 0.03% DDM to remove contaminants. Protein was concentrated to 32 mg/ml using centrifugal concentrators (Sartorius) with a relative molecular mass cut‐off of 100 K.

### Transport time course

CodB was expressed in Lemo21(DE3) cells as above with 25 ml culture volumes. Following centrifugation of the cultures at 2,600 *g* for 10 min at 20°C, the supernatant was removed and the pellet was resuspended in 5 ml 5 mM MES pH 6.6, 150 mM KCl. This was repeated three times. Cells were resuspended to give a final concentration of OD_600_ of 2 in 1,200 μl of either 5 mM MES pH 6.6, 150 mM NaCl or 5 mM MES pH 6.6, 150 mM choline chloride. 6 μl of 6.25 μM ^3^H‐5‐cytosine (20 Ci mmol^−1^; American Radiolabelled Chemicals) was added to samples and the cells were incubated at 37°C with shaking at 900 rpm for times of 30 s, 1, 2, 5, 10, or 20 min. At the stated timepoint, 200 μl of cells were centrifuged at 16,000 *g* for 30 s at 20°C, the supernatant was removed and the pellet was resuspended in 200 μl stop buffer (5 mM MES pH 6.6, 150 mM KCl, 1 mM cytosine) and added to a 0.2 μm Whatman cellulose nitrate membrane filter under vacuum followed by immediate washing with 4 × 2 ml 0.1 M LiCl. Each filter was placed in 10 ml Emulsifier Safe scintillation fluid and counted using a Tri‐CarbA4810TR Liquid Scintillation Analyzer (Perkin Elmer). CodB concentration was quantified based on the GFP fluorescence. Lemo21 cells with no CodB overexpression were used as a background, with 1 μl of 6.25 μM ^3^H‐5‐cytosine used to calibrate counts. Experiments were performed in triplicate with fresh cultures.

### Inhibition assay

Cells were prepared as described previously and resuspended to an OD600 of 2 in 200 μl to give a final concentration with 5 mM MES pH 6.6, 150 mM NaCl, 0.1 mM potential inhibitor. 1 μl of 6.25 μM ^3^H‐5‐cytosine was added and the mixture incubated at 37°C with shaking at 900 rpm for 1 min before centrifuging at 16,000 *g* for 1 min at 20°C. The supernatant was removed and the pellet was resuspended in 200 μl stop buffer (5 mM MES pH 6.6, 150 mM KCl, 1 mM cytosine) and added to a 0.2 μm Whatman cellulose nitrate membrane filter under vacuum followed by immediate washing with 4 × 2 ml 0.1 M LiCl. All filters were dissolved in 10 ml Emulsifier Safe scintillation fluid and counted using a Tri‐Carb A4810TR Liquid Scintillation Analyzer (Perkin Elmer). Experiments were performed in triplicate with fresh cultures.

### Activity assay for mutants

Mutants were tested for activity using a similar assay to the inhibition assay using a 1 min timepoint. Each assay was run by testing mutant and wild‐type expressing cultures in parallel. The cultures were resuspended to an OD of 2.0 as above and the results from the scintillation counting corrected for the slight differences in expression level of the protein as judged from the fluorescence counts associated with the GFP. Replicates were from freshly prepared cultures.

### GFP‐TS

The GFP‐TS assay was carried out following the published protocol (Nji *et al*, 2018). 150 μl of *E. coli* membrane with overexpressed CodB was diluted 1:10 in 20 mM TRIS pH 7.5, 150 mM NaCl, 1% DDM, 1% octyl‐β‐D‐glucoside, (β‐OG), 1 mM of the molecule to be tested and left mixing at 4°C for 1 h then aliquoted into 150 μl fractions. Aliquots were subjected to various temperatures, 4, 20, 25, 30, 35, 40, 45, 50, 60°C for 10 min then spun at 16,000 *g* for 30 min. 100 μl of supernatant was transferred to a 96‐well black‐walled plate and GFP measurements were taken. The apparent T_m_ for each titration was calculated by plotting the normalised average GFP fluorescence intensity from two technical repeats at each temperature and fitting the curves to a sigmoidal dose–response equation (variable slope) by GraphPad Prism software (version 9.0). Values reported are the averaged mean of the fit from *n* = 2 independent titrations.

To generate an approximate K_d_ 150 μl of *E. coli* membrane was solubilised as before but cytosine was added at a final concentration between 0 and 1,000 μM. Aliquots were put at 35°C for 10 min and spun at 16,000 *g* for 30 min. 100 μl of supernatant was transferred to a 96‐well black plate and GFP measurements were taken. The binding curve was fitted by nonlinear regression (one site, total binding) by GraphPad Prism software (version 9.0), and the values reported are the averaged mean ± s.e.m. of the fit from *n* = 3 independent titrations.

### Crystallisation and structural determination

Protein at a concentration of 32 mg/ml was subjected to crystallisation using the lipidic cubic phase method of crystallisation (Caffrey & Cherezov, 2009). The CodB protein with 1 mM cytosine was mixed with monoolein at 60:40 (w/w) ratio using a coupled syringe device (SPT Labtech) and crystallisation trials were set up at 20°C using glass sandwich plates using a Mosquito Robot. Crystals appeared in condition G5 of MemGoldMeso (Molecular Dimensions Ltd) in glass sandwich plates, which contained 0.1 M sodium cacodylate pH 6.5, 0.45 M NaCl, 39% PEG400. Crystals were cryo‐cooled in liquid nitrogen.

X‐ray diffraction data were collected at beamline I24 at Diamond Light Source, UK. Initially, a data set was collected that was processed at 3.6 Å resolution but subsequently a higher resolution data set was collected. Data were processed using DIALS (Waterman *et al*, 2016) through the Xia2 pipeline (Winter *et al*, 2013). Processed data were then scaled and merged in AIMLESS (Evans & Murshudov, 2013) in the CCP4 suite (Collaborative Computational Project Number 4, 1994). The resolution cut‐off was chosen based on where the CC_0.5_ fell below 0.5. The structure was solved from the 3.6 Å resolution data set using MR_ROSETTA (DiMaio *et al*, 2011) in the PHENIX package (Liebschner *et al*, 2019) basing the search on the outward‐facing structure of Mhp1 (2JLN; Weyand *et al*, 2008). Refinement was carried out with PHENIX.REFINE (Afonine *et al*, 2012) interspersed with model building in Coot (Emsley & Cowtan, 2004) initially against the low‐resolution data set but subsequently against the high‐resolution data set. Table 1 was calculated with PHENIX.

Superpositions were performed in Chimera (Pettersen *et al*, 2004) maintaining the default cut‐off of 2 Å for pruning matching C_α_ atoms and structural images were prepared in PyMol (Delano, 2002). Images involving electron density were prepared in CCP4mg (McNicholas *et al*, 2011) except Appendix Fig S2, which was made with Chimera.

To obtain the sequence alignment for proteins similar to CodB from *P. vulgaris* a BLAST search (Altschul *et al*, 1990) was carried out at the EBI (Madeira *et al*, 2019) against the Uniref90 database from the Uniref clusters (Suzek *et al*, 2015) selecting 200 sequences. These were aligned using Clustal Omega (Sievers *et al*, 2011) and imported into Jalview (Waterhouse *et al*, 2009). Similar sequences were removed using the “Remove Redundancy” tool in Jalview. The sequence alignment figure was based on the image output from Jalview.

## Data availability

The structure and data have been deposited in the RCSB with accession number 7QOA (https://www.rcsb.org/structure/7QOA).

## Author contributions

**Caitlin E Hatton:** Investigation; writing – original draft; writing – review and editing. **Deborah H Brotherton:** Supervision; investigation; writing – review and editing. **Mahalah Spencer:** Investigation. **Alexander D Cameron:** Conceptualization; supervision; investigation; writing – original draft; writing – review and editing.

In addition to the CRediT author contributions listed above, the contributions in detail are:

The project was initiated by AC. CEH carried out most experiments, with help and supervision from AC and DHB. MS contributed to the GFP‐TS assays. The paper was written by AC with contributions from CEH and DHB.

## Disclosure and competing interests statement

The authors declare that they have no conflict of interest.

## Supporting information



Appendix S1

Expanded View Figures PDF

## References

[embj2021110527-bib-0001] Abramson J , Wright EM (2009) Structure and function of Na(+)‐symporters with inverted repeats. Curr Opin Struct Biol 19: 425–432 19631523 10.1016/j.sbi.2009.06.002 PMC3496787

[embj2021110527-bib-0002] Afonine PV , Grosse‐Kunstleve RW , Echols N , Headd JJ , Moriarty NW , Mustyakimov M , Terwilliger TC , Urzhumtsev A , Zwart PH , Adams PD (2012) Towards automated crystallographic structure refinement with phenix.Refine. Acta Crystallogr D Biol Crystallogr 68: 352–367 22505256 10.1107/S0907444912001308 PMC3322595

[embj2021110527-bib-0003] Altschul SF , Gish W , Miller W , Myers EW , Lipman DJ (1990) Basic local alignment search tool. J Mol Biol 215: 403–410 2231712 10.1016/S0022-2836(05)80360-2

[embj2021110527-bib-0004] Caffrey M , Cherezov V (2009) Crystallizing membrane proteins using lipidic mesophases. Nat Protoc 4: 706–731 19390528 10.1038/nprot.2009.31 PMC2732203

[embj2021110527-bib-0005] Calabrese AN , Jackson SM , Jones LN , Beckstein O , Heinkel F , Gsponer J , Sharples D , Sans M , Kokkinidou M , Pearson AR *et al* (2017) Topological dissection of the membrane transport protein Mhp1 derived from cysteine accessibility and mass spectrometry. Anal Chem 89: 8844–8852 28726379 10.1021/acs.analchem.7b01310 PMC5588088

[embj2021110527-bib-0006] Coleman JA , Green EM , Gouaux E (2016) X‐ray structures and mechanism of the human serotonin transporter. Nature 532: 334–339 27049939 10.1038/nature17629 PMC4898786

[embj2021110527-bib-0007] Coleman JA , Yang D , Zhao Z , Wen PC , Yoshioka C , Tajkhorshid E , Gouaux E (2019) Serotonin transporter‐ibogaine complexes illuminate mechanisms of inhibition and transport. Nature 569: 141–145 31019304 10.1038/s41586-019-1135-1 PMC6750207

[embj2021110527-bib-0008] Collaborative Computational Project Number 4 (1994) The CCP4 suite: Programs for protein crystallography. Acta Crystallogr D Biol Crystallogr 50: 760–763 15299374 10.1107/S0907444994003112

[embj2021110527-bib-0009] Danielsen S , Kilstrup M , Barilla K , Jochimsen B , Neuhard J (1992) Characterization of the Escherichia coli codBA operon encoding cytosine permease and cytosine deaminase. Mol Microbiol 6: 1335–1344 1640834 10.1111/j.1365-2958.1992.tb00854.x

[embj2021110527-bib-0010] Delano WL (2002) The PyMOL molecular graphics system. Palo Alto, CA: DeLano Scientific

[embj2021110527-bib-0011] DiMaio F , Terwilliger TC , Read RJ , Wlodawer A , Oberdorfer G , Wagner U , Valkov E , Alon A , Fass D , Axelrod HL *et al* (2011) Improved molecular replacement by density‐ and energy‐guided protein structure optimization. Nature 473: 540–543 21532589 10.1038/nature09964 PMC3365536

[embj2021110527-bib-0012] Drew D , Lerch M , Kunji E , Slotboom DJ , de Gier JW (2006) Optimization of membrane protein overexpression and purification using GFP fusions. Nat Methods 3: 303–313 16554836 10.1038/nmeth0406-303

[embj2021110527-bib-0013] Edgar RC (2004) MUSCLE: Multiple sequence alignment with high accuracy and high throughput. Nucleic Acids Res 32: 1792–1797 15034147 10.1093/nar/gkh340 PMC390337

[embj2021110527-bib-0014] Emsley P , Cowtan K (2004) Coot: Model‐building tools for molecular graphics. Acta Crystallogr D Biol Crystallogr 60: 2126–2132 15572765 10.1107/S0907444904019158

[embj2021110527-bib-0015] Evans PR , Murshudov GN (2013) How good are my data and what is the resolution? Acta Crystallogr D Biol Crystallogr 69: 1204–1214 23793146 10.1107/S0907444913000061 PMC3689523

[embj2021110527-bib-0016] Faham S , Watanabe A , Besserer GM , Cascio D , Specht A , Hirayama BA , Wright EM , Abramson J (2008) The crystal structure of a sodium galactose transporter reveals mechanistic insights into Na+/sugar symport. Science 321: 810–814 18599740 10.1126/science.1160406 PMC3654663

[embj2021110527-bib-0017] Focht D , Neumann C , Lyons J , Eguskiza Bilbao A , Blunck R , Malinauskaite L , Schwarz IO , Javitch JA , Quick M , Nissen P (2021) A non‐helical region in transmembrane helix 6 of hydrophobic amino acid transporter MhsT mediates substrate recognition. EMBO J 40: e105164 33155685 10.15252/embj.2020105164 PMC7780149

[embj2021110527-bib-0018] Forrest LR , Zhang YW , Jacobs MT , Gesmonde J , Xie L , Honig BH , Rudnick G (2008) Mechanism for alternating access in neurotransmitter transporters. Proc Natl Acad Sci U S A 105: 10338–10343 18647834 10.1073/pnas.0804659105 PMC2480614

[embj2021110527-bib-0019] Han L , Qu Q , Aydin D , Panova O , Robertson MJ , Xu Y , Dror RO , Skiniotis G , Feng L (2022) Structure and mechanism of the SGLT family of glucose transporters. Nature 601: 274–279 34880492 10.1038/s41586-021-04211-w PMC9482448

[embj2021110527-bib-0020] Imperi F , Massai F , Facchini M , Frangipani E , Visaggio D , Leoni L , Bragonzi A , Visca P (2013) Repurposing the antimycotic drug flucytosine for suppression of Pseudomonas aeruginosa pathogenicity. Proc Natl Acad Sci U S A 110: 7458–7463 23569238 10.1073/pnas.1222706110 PMC3645532

[embj2021110527-bib-0021] Jardetzky O (1966) Simple allosteric model for membrane pumps. Nature 211: 969–970 5968307 10.1038/211969a0

[embj2021110527-bib-0022] Kazmier K , Sharma S , Islam SM , Roux B , McHaourab HS (2014a) Conformational cycle and ion‐coupling mechanism of the Na+/hydantoin transporter Mhp1. Proc Natl Acad Sci U S A 111: 14752–14757 25267652 10.1073/pnas.1410431111 PMC4205665

[embj2021110527-bib-0023] Kazmier K , Sharma S , Quick M , Islam SM , Roux B , Weinstein H , Javitch JA , McHaourab HS (2014b) Conformational dynamics of ligand‐dependent alternating access in LeuT. Nat Struct Mol Biol 21: 472–479 24747939 10.1038/nsmb.2816 PMC4050370

[embj2021110527-bib-0024] Kniazeff J , Shi L , Loland CJ , Javitch JA , Weinstein H , Gether U (2008) An intracellular interaction network regulates conformational transitions in the dopamine transporter. J Biol Chem 283: 17691–17701 18426798 10.1074/jbc.M800475200 PMC2427322

[embj2021110527-bib-0025] de Koning H , Diallinas G (2000) Nucleobase transporters (review). Mol Membr Biol 17: 75–94 10989458 10.1080/09687680050117101

[embj2021110527-bib-0026] Krishnamurthy H , Gouaux E (2012) X‐ray structures of LeuT in substrate‐free outward‐open and apo inward‐open states. Nature 481: 469–474 22230955 10.1038/nature10737 PMC3306218

[embj2021110527-bib-0027] Lee C , Kang HJ , Hjelm A , Qureshi AA , Nji E , Choudhury H , Beis K , de Gier JW , Drew D (2014) MemStar: A one‐shot Escherichia coli‐based approach for high‐level bacterial membrane protein production. FEBS Lett 588: 3761–3769 25176409 10.1016/j.febslet.2014.08.025

[embj2021110527-bib-0028] Liebschner D , Afonine PV , Baker ML , Bunkoczi G , Chen VB , Croll TI , Hintze B , Hung LW , Jain S , McCoy AJ *et al* (2019) Macromolecular structure determination using X‐rays, neutrons and electrons: Recent developments in phenix. Acta Crystallogr D Struct Biol 75: 861–877 31588918 10.1107/S2059798319011471 PMC6778852

[embj2021110527-bib-0029] Ma P , Varela F , Magoch M , Silva AR , Rosario AL , Brito J , Oliveira TF , Nogly P , Pessanha M , Stelter M *et al* (2013) An efficient strategy for small‐scale screening and production of archaeal membrane transport proteins in Escherichia coli. PLoS One 8: e76913 24282478 10.1371/journal.pone.0076913 PMC3838208

[embj2021110527-bib-0030] Madeira F , Park YM , Lee J , Buso N , Gur T , Madhusoodanan N , Basutkar P , Tivey ARN , Potter SC , Finn RD *et al* (2019) The EMBL‐EBI search and sequence analysis tools APIs in 2019. Nucleic Acids Res 47: W636–W641 30976793 10.1093/nar/gkz268 PMC6602479

[embj2021110527-bib-0031] Malinauskaite L , Quick M , Reinhard L , Lyons JA , Yano H , Javitch JA , Nissen P (2014) A mechanism for intracellular release of Na+ by neurotransmitter/sodium symporters. Nat Struct Mol Biol 21: 1006–1012 25282149 10.1038/nsmb.2894 PMC4346222

[embj2021110527-bib-0032] Maura D , Ballok AE , Rahme LG (2016) Considerations and caveats in anti‐virulence drug development. Curr Opin Microbiol 33: 41–46 27318551 10.1016/j.mib.2016.06.001 PMC5069136

[embj2021110527-bib-0033] McNicholas S , Potterton E , Wilson KS , Noble ME (2011) Presenting your structures: The CCP4mg molecular‐graphics software. Acta Crystallogr D Biol Crystallogr 67: 386–394 21460457 10.1107/S0907444911007281 PMC3069754

[embj2021110527-bib-0034] Mourad GS , Tippmann‐Crosby J , Hunt KA , Gicheru Y , Bade K , Mansfield TA , Schultes NP (2012) Genetic and molecular characterization reveals a unique nucleobase cation symporter 1 in Arabidopsis. FEBS Lett 586: 1370–1378 22616996 10.1016/j.febslet.2012.03.058

[embj2021110527-bib-0035] Muse WB , Rosario CJ , Bender RA (2003) Nitrogen regulation of the codBA (cytosine deaminase) operon from Escherichia coli by the nitrogen assimilation control protein, NAC. J Bacteriol 185: 2920–2926 12700271 10.1128/JB.185.9.2920-2926.2003 PMC154391

[embj2021110527-bib-0036] Niu Y , Liu R , Guan C , Zhang Y , Chen Z , Hoerer S , Nar H , Chen L (2022) Structural basis of inhibition of the human SGLT2–MAP17 glucose transporter. Nature 601: 280–284 34880493 10.1038/s41586-021-04212-9

[embj2021110527-bib-0037] Nji E , Chatzikyriakidou Y , Landreh M , Drew D (2018) An engineered thermal‐shift screen reveals specific lipid preferences of eukaryotic and prokaryotic membrane proteins. Nat Commun 9: 4253 30315156 10.1038/s41467-018-06702-3 PMC6185904

[embj2021110527-bib-0038] Pantazopoulou A , Diallinas G (2007) Fungal nucleobase transporters. FEMS Microbiol Rev 31: 657–675 17784857 10.1111/j.1574-6976.2007.00083.x

[embj2021110527-bib-0039] Papadaki GF , Lambrinidis G , Zamanos A , Mikros E , Diallinas G (2019) Cytosolic N‐ and C‐termini of the aspergillus nidulans FurE transporter contain distinct elements that regulate by long‐range effects function and specificity. J Mol Biol 431: 3827–3844 31306663 10.1016/j.jmb.2019.07.013

[embj2021110527-bib-0040] Penmatsa A , Wang KH , Gouaux E (2013) X‐ray structure of dopamine transporter elucidates antidepressant mechanism. Nature 503: 85–90 24037379 10.1038/nature12533 PMC3904663

[embj2021110527-bib-0041] Penmatsa A , Wang KH , Gouaux E (2015) X‐ray structures of drosophila dopamine transporter in complex with nisoxetine and reboxetine. Nat Struct Mol Biol 22: 506–508 25961798 10.1038/nsmb.3029 PMC4608549

[embj2021110527-bib-0042] Perez C , Koshy C , Yildiz O , Ziegler C (2012) Alternating‐access mechanism in conformationally asymmetric trimers of the betaine transporter BetP. Nature 490: 126–130 22940865 10.1038/nature11403

[embj2021110527-bib-0043] Pettersen EF , Goddard TD , Huang CC , Couch GS , Greenblatt DM , Meng EC , Ferrin TE (2004) UCSF Chimera‐a visualization system for exploratory research and analysis. J Comput Chem 25: 1605–1612 15264254 10.1002/jcc.20084

[embj2021110527-bib-0044] Ressl S , Terwisscha van Scheltinga AC , Vonrhein C , Ott V , Ziegler C (2009) Molecular basis of transport and regulation in the Na(+)/betaine symporter BetP. Nature 458: 47–52 19262666 10.1038/nature07819

[embj2021110527-bib-0045] Santos‐Zavaleta A , Salgado H , Gama‐Castro S , Sanchez‐Perez M , Gomez‐Romero L , Ledezma‐Tejeida D , Garcia‐Sotelo JS , Alquicira‐Hernandez K , Muniz‐Rascado LJ , Pena‐Loredo P *et al* (2019) RegulonDB v 10.5: Tackling challenges to unify classic and high throughput knowledge of gene regulation in E. coli K‐12. Nucleic Acids Res 47: D212–D220 30395280 10.1093/nar/gky1077 PMC6324031

[embj2021110527-bib-0046] Schein JR , Hunt KA , Minton JA , Schultes NP , Mourad GS (2013) The nucleobase cation symporter 1 of Chlamydomonas reinhardtii and that of the evolutionarily distant Arabidopsis thaliana display parallel function and establish a plant‐specific solute transport profile. Plant Physiol Biochem 70: 52–60 23770594 10.1016/j.plaphy.2013.05.015

[embj2021110527-bib-0047] Shahsavar A , Stohler P , Bourenkov G , Zimmermann I , Siegrist M , Guba W , Pinard E , Sinning S , Seeger MA , Schneider TR *et al* (2021) Structural insights into the inhibition of glycine reuptake. Nature 591: 677–681 33658720 10.1038/s41586-021-03274-z

[embj2021110527-bib-0048] Shimamura T , Weyand S , Beckstein O , Rutherford NG , Hadden JM , Sharpies D , Sansom MSP , Iwata S , Henderson PJF , Cameron AD (2010) Molecular basis of alternating access membrane transport by the sodium‐hydantoin transporter Mhp1. Science 328: 470–473 20413494 10.1126/science.1186303 PMC2885435

[embj2021110527-bib-0050] Sievers F , Wilm A , Dineen D , Gibson TJ , Karplus K , Li W , Lopez R , McWilliam H , Remmert M , Soding J *et al* (2011) Fast, scalable generation of high‐quality protein multiple sequence alignments using Clustal omega. Mol Syst Biol 7: 539 21988835 10.1038/msb.2011.75 PMC3261699

[embj2021110527-bib-0051] Simmons KJ , Jackson SM , Brueckner F , Patching SG , Beckstein O , Ivanova E , Geng T , Weyand S , Drew D , Lanigan J *et al* (2014) Molecular mechanism of ligand recognition by membrane transport protein, Mhp1. EMBO J 33: 1831–1844 24952894 10.15252/embj.201387557 PMC4195764

[embj2021110527-bib-0052] Sonoda Y , Newstead S , Hu NJ , Alguel Y , Nji E , Beis K , Yashiro S , Lee C , Leung J , Cameron AD *et al* (2011) Benchmarking membrane protein detergent stability for improving throughput of high‐resolution X‐ray structures. Structure 19: 17–25 21220112 10.1016/j.str.2010.12.001 PMC3111809

[embj2021110527-bib-0053] Suzek BE , Wang Y , Huang H , McGarvey PB , Wu CH , UniProt Consortium (2015) UniRef clusters: A comprehensive and scalable alternative for improving sequence similarity searches. Bioinformatics 31: 926–932 25398609 10.1093/bioinformatics/btu739 PMC4375400

[embj2021110527-bib-0054] Vermes A , Guchelaar HJ , Dankert J (2000) Flucytosine: A review of its pharmacology, clinical indications, pharmacokinetics, toxicity and drug interactions. J Antimicrob Chemother 46: 171–179 10933638 10.1093/jac/46.2.171

[embj2021110527-bib-0055] Wahlgren WY , Dunevall E , North RA , Paz A , Scalise M , Bisignano P , Bengtsson‐Palme J , Goyal P , Claesson E , Caing‐Carlsson R *et al* (2018) Substrate‐bound outward‐open structure of a Na(+)‐coupled sialic acid symporter reveals a new Na(+) site. Nat Commun 9: 1753 29717135 10.1038/s41467-018-04045-7 PMC5931594

[embj2021110527-bib-0056] Waterhouse AM , Procter JB , Martin DM , Clamp M , Barton GJ (2009) Jalview version 2‐‐a multiple sequence alignment editor and analysis workbench. Bioinformatics 25: 1189–1191 19151095 10.1093/bioinformatics/btp033 PMC2672624

[embj2021110527-bib-0057] Waterman DG , Winter G , Gildea RJ , Parkhurst JM , Brewster AS , Sauter NK , Evans G (2016) Diffraction‐geometry refinement in the DIALS framework. Acta Crystallogr D Struct Biol 72: 558–575 27050135 10.1107/S2059798316002187 PMC4822564

[embj2021110527-bib-0058] Weyand S , Shimamura T , Yajima S , Suzuki S , Mirza O , Krusong K , Carpenter EP , Rutherford NG , Hadden JM , O'Reilly J *et al* (2008) Structure and molecular mechanism of a nucleobase‐cation‐symport‐1 family transporter. Science 322: 709–713 18927357 10.1126/science.1164440 PMC2885439

[embj2021110527-bib-0059] Winter G , Lobley CM , Prince SM (2013) Decision making in xia2. Acta Crystallogr D Biol Crystallogr 69: 1260–1273 23793152 10.1107/S0907444913015308 PMC3689529

[embj2021110527-bib-0060] Witz S , Panwar P , Schober M , Deppe J , Pasha FA , Lemieux MJ , Mohlmann T (2014) Structure‐function relationship of a plant NCS1 member‐‐homology modeling and mutagenesis identified residues critical for substrate specificity of PLUTO, a nucleobase transporter from *Arabidopsis* . PLoS One 9: e91343 24621654 10.1371/journal.pone.0091343 PMC3951388

[embj2021110527-bib-0061] Wong FH , Chen JS , Reddy V , Day JL , Shlykov MA , Wakabayashi ST , Saier MH Jr (2012) The amino acid‐polyamine‐organocation superfamily. J Mol Microbiol Biotechnol 22: 105–113 22627175 10.1159/000338542

[embj2021110527-bib-0062] Yamashita A , Singh SK , Kawate T , Jin Y , Gouaux E (2005) Crystal structure of a bacterial homologue of Na+/Cl‐‐dependent neurotransmitter transporters. Nature 437: 215–223 16041361 10.1038/nature03978

