# Supplementary material for: Structure of cytosine transport protein CodB provides insight into nucleobase‐cation symporter 1 mechanism
Source: EMBO J. 2022 Jul 1;41(16):e110527. doi: 10.15252/embj.2021110527 (PMC9379551; doi:10.15252/embj.2021110527)
Supplement: Supplementary file 1 — Appendix S1 [file EMBJ-41-e110527-s001.pdf]

Appendix for

Structure of cytosine transport protein CodB provides insight into nucleobase-cation symporter 1 mechanism

Caitlin E. Hatton[1], Deborah H. Brotherton, Mahalah Spencer, Alexander D. Cameron*

Table of Contents:

Appendix Table S1
Appendix Figures S1-S4 (including figure legends)
Sequence Information

Mutation	Forward (top) and Reverse (bottom) primer
Q105A	5' CTGGGGGGGACTGCGGTTGGATGGTTTGGTGTAG 3'<br>5' CTACACCAAACCATCCAACCGCAGTCCCCCCCAG 3'
W108A	5' GGGACTCAGGTTGGAGCGTTTGGTGTAGGCGTCGCTATG 3'<br>5' CATAGCGACGCCTACACCAAACGCTCCAACCTGAGTCCC 3'
F204A	5' CGATGGTCGTGGGATCAGCCATTAGCGCTGGTACACTTAC 3'<br>5' GTAAGTGTACCAGCGCTAATGGCTGATCCCACGACCATCG 3'
T278A	5' CTGGGGTTTGAATATTTGGGCCACAAATGACAATGCC 3'<br>5' GGCATTGTCATTTGTGGCCCAAATATTCAAACCCCAG 3'
T279A	5' CTGGGGTTTGAATATTTGGACCGCAAATGACAATGCCCTTTACG 3'<br>5' CGTAAAGGGCATTGTCATTTGCGGTCCAAATATTCAAACCCCAG 3'
T278AT279A	5' CTGGGTTTGAATATTTGGGCCGCAAATGACAATGCC 3'<br>5' GGCATTGTCATTTGCGGCCCAAATATTCAAACCCAG 3'
N280A	5' GTCCTGGGTTTGAATATTTGGACCACAGCTGACAATGCCCTTTAC 3'<br>5' GTAAAGGGCATTGTCAGCTGTGGTCCAAATATTCAAACCCAGGAC 3'

Appendix Table S1: Primers used to create mutants.

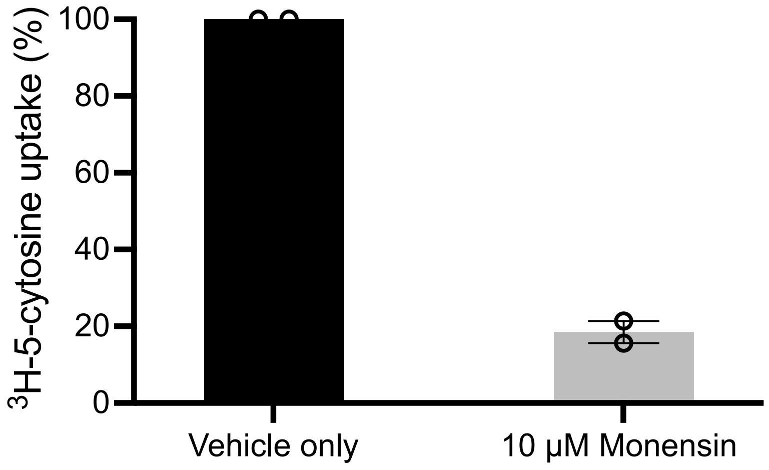

Appendix Figure S1. Uptake of ^{3}H-5-cytosine in the presence of the sodium ionophore Monensin. Uptake of ^{3}H-5-cytosine was measured after 1 minute. Uptake for the protein with just the vehicle in which the monensin was dissolved was set at 100%, and the data from the monensin containing experiments scaled accordingly. The results from 2 independent measurements are shown.

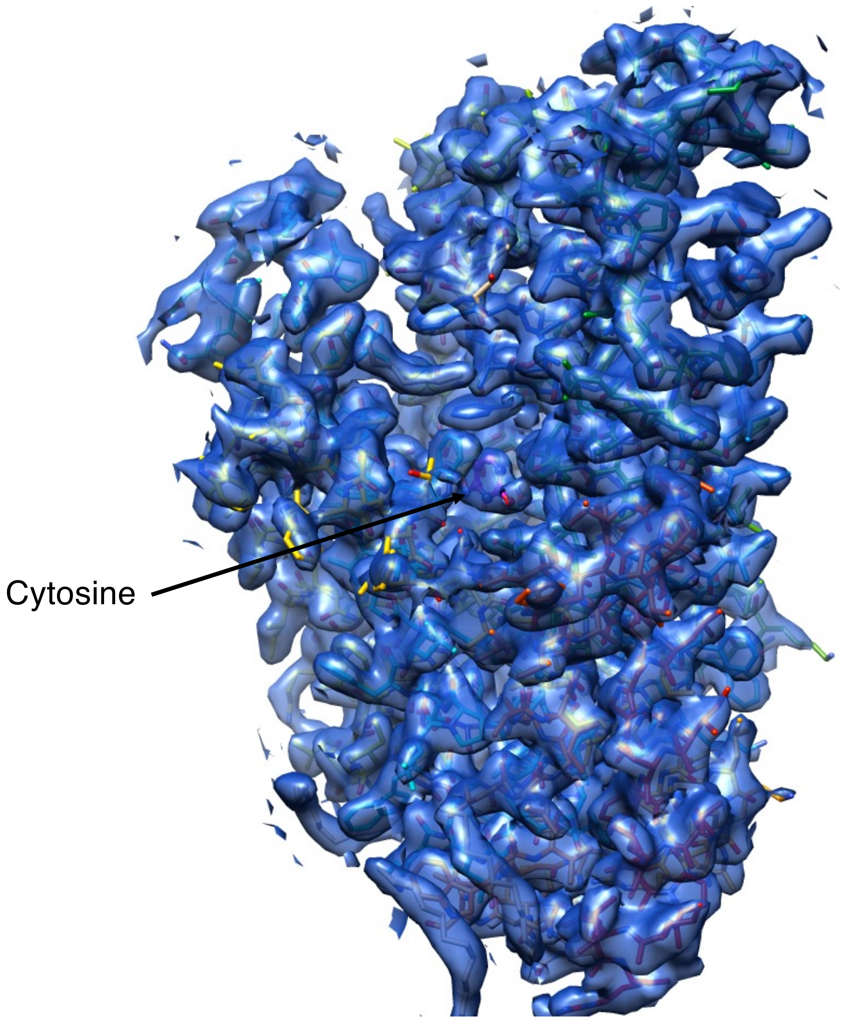

Appendix Figure S2. Refined electron density associated with one molecule of the asymmetric unit. The 2mFo-DFc map is shown as a semi-transparent surface with the refined structures below.

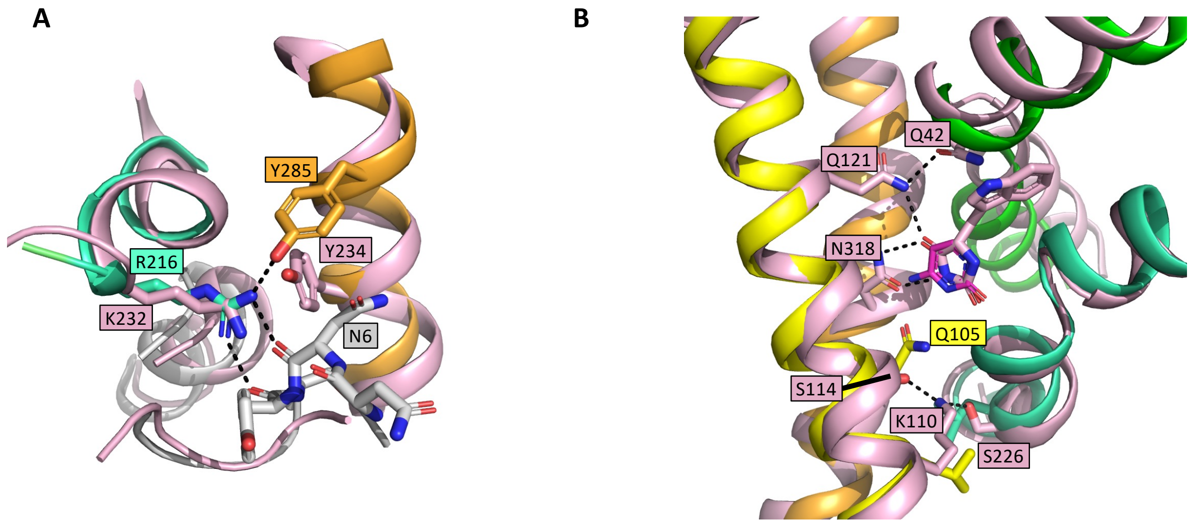

Appendix Figure S3. Interaction between residues of the bundle and hash domains in CodB and Mhp1. **A)** Interaction between Arg216 and Tyr285 coloured as shown in Fig EV5C with Mhp1 superposed. In Mhp1 Arg216 is replaced by a lysine and while Tyr285 is not conserved, the hydroxyl group of another tyrosine could interact with the lysine. **B)** Comparison between the hydrogen bonding arrangement in CodB and Mhp1 with potential hydrogen bonds shown for Mhp1

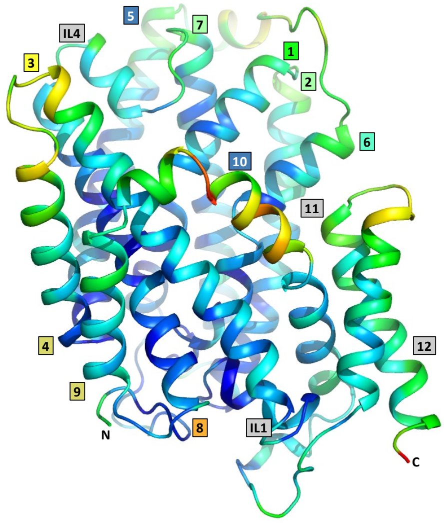

Appendix Figure S4. The structure of CodB coloured by temperature factors. The higher the temperature factor the warmer the colour.

Sequence Information

Sequence of CodB up to 3C protease site. Bases extra to CodB that would be expressed in the modified pWaldo vector are shown in italics.

ATGAGCCAGGACAACAACTATAGTCAAGGGCCAGTCCCTATTTCCGCACGTAAGGGAGGACTTGCC
CTTACCTTTGTCATGTTAGGTCTGACATTCTTTTCCGCATCTATGTGGACCGGAGGCGCTCTTGGGAC
AGGACTGTCGTTCAACGACTTTTTCCTTGCGGTTCTGATCGGAAACCTGCTTCTGGGTATCTACACGG
CCTTCCTGGGTTTTATCGGGAGTAAGACTGGGTTAACTACTCACTTGCTTGCCCGTTACTCGTTCGGC
ATCAAAGGGTCCTGGTTACCCTCATTTCTTCTGGGGGGGACTCAGGTTGGATGGTTTGGTGTAGGCG
TCGCTATGTTTGCGATTCCGGTGGGTAAAGCCACGGGAATTGACATCAACTTACTGATTGCTGTTAG
TGGGATTCTGATGACCATTACTGTGTTCTTTGGTATCTCTGCGCTGACCGTTTTATCCATCATCGCCGT
TCCGGCTATCGCAATCCTTGGCAGTTATAGCGTTTATCTGGCGATCCACGACATGGGCGGGCTGAGC
ACGCTTATGAACGTGAAGCCCACACAACCATTAGACTTTAATTTAGCCCTTGCGATGGTCGTGGGAT
CATTCATTAGCGCTGGTACACTTACAGCCGATTTCGTCCGTTTCGGTCGTAACCCAAAAGTTGCAGTC
GTTGTGGCAATCATCGCTTTCTTTTTAGGCAATACGCTTATGTTTGTATTTGGCGCGGCTGGGGCCGC
GTCGTTGGGAATGGCCGACATCTCTGATGTCATGATCGCTCAGGGGTTACTTCTGCCGGCTATCGTG
GTCCTGGGTTTGAATATTTGGACCACAAATGACAATGCCCTTTACGCCTCAGGATTAGGTTTTGCAAA
CATTACCGGGTTGAGTAGCAAGAAGTTGAGCGTGATCAACGGCATCGTTGGAACGGTGTGTGCTCT
GTGGTTGTACAACAATTTCGTTGGTTGGCTTACATTCCTGTCAGCAGCAATCCCGCCTGTCGGTGGG
GTTATTATCGCCGACTATCTTATGAATAAGGCACGCTATAACACTTTTAATATCGCAACCATGCAGTC
CGTCAATTGGGTAGCTTTGCTGGCCGTGGCTATTGGCATCGTAGCGGGCCATTGGTTGCCGGGCATT
GTGCCTGTTAACGCCGTTTTAGGCGGCGCCATTAGCTACGCAGTTTTAAATCCGATCTTAAATCGCCG
CACCGCACGCCAGGCTGAAATTTCTCATGCTGGA*TCCCTGGAAGTCCTGTTCCAGGGTCCGTTC*

Corresponding protein sequence with the 3C protease site underlined. The sequence of CodB is shown in bold. The residues that remain after cleavage by 3C protease are shown in italics.

MSQDNNYSQGPVPISARKGGLALTFVMLGLTFFSASMWTGGALGTGLSFNDFFLAVLIGNLLLGIYTA
FLGFIGSKTGLTTHLLARYSFGIKGSWLPSFLLGGTQVGWFGVGVAMFAIPVGKATGIDINLLIAVSGIL
MTITVFFGISALTVLSIIAVPAIAILGSYSVYLAIHDMGGLSTLMNVKPTQPLDFNLALAMVVGSFISAGT
LTADFVRFGRNPKVAVVVAIIAFFLGNTLMFVFGAAGAASLGMADISDVMIAQGLLLPAIVVLGLNIW
TTNDNALYASGLGFANITGLSSKKLSVINGIVGTVCALWLYNNFVGWLTFLSAAIPPVGGVIIADYLMN
KARYNTFNIATMQSVNWVALLAVAIGIVAGHWLPGIVPVNAVLGGAISYAVLNPILNRRTARQAEISH
AG*SLEVLFQ*GPF
