# Supplementary material for: Structure of cytosine transport protein CodB provides insight into nucleobase‐cation symporter 1 mechanism
Source: EMBO J. 2022 Jul 1;41(16):e110527. doi: 10.15252/embj.2021110527 (PMC9379551; doi:10.15252/embj.2021110527)
Supplement: Supplementary file 2 — Expanded View Figures PDF [file EMBJ-41-e110527-s003.pdf]

Expanded View Figures

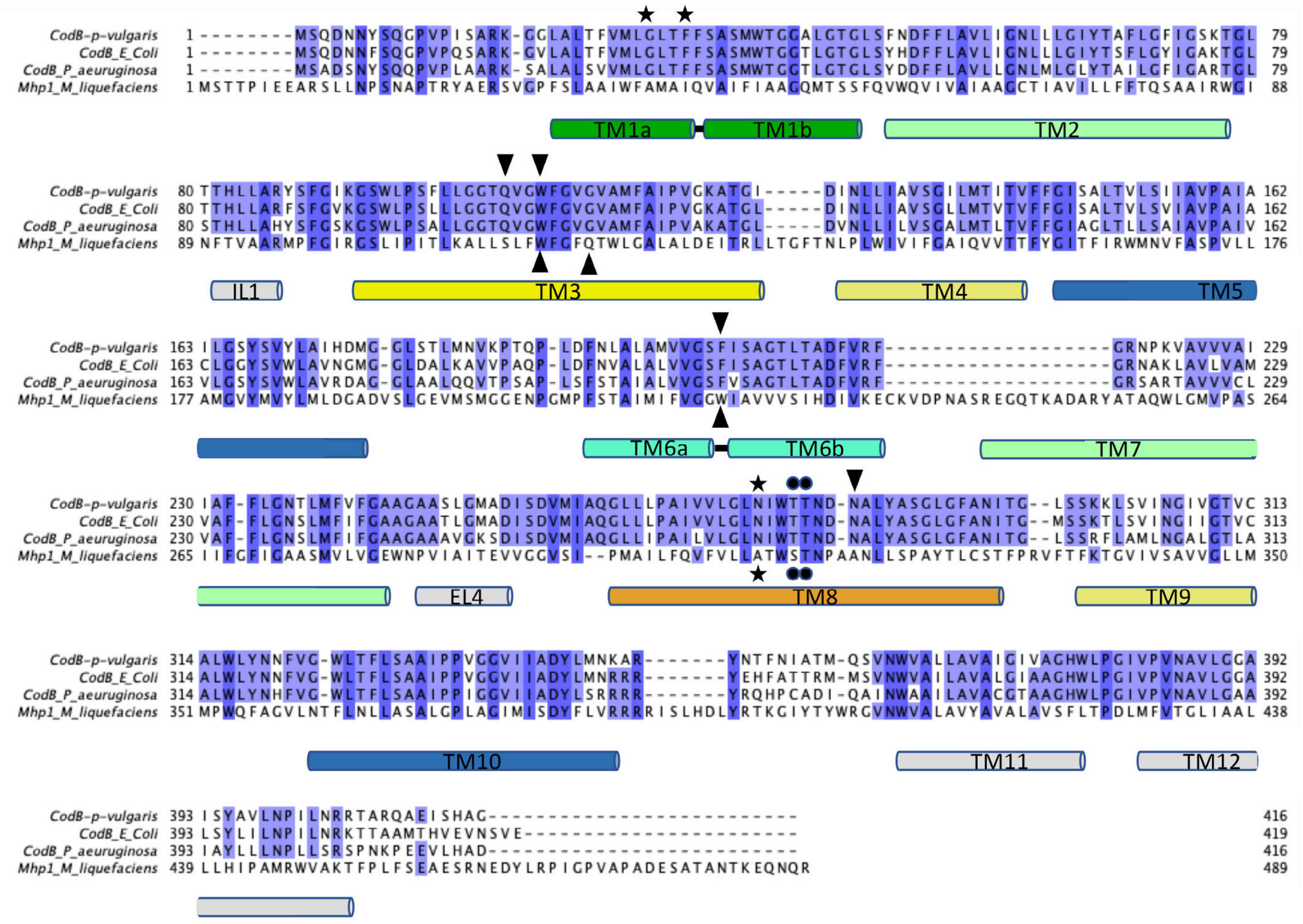

Figure EV1. Sequence alignment of CodB and Mhp1.

Alignment of sequences of CodB from *P. vulgaris*, *E. coli* and *P. aeruginosa* with Mhp1 from *M. liquefaciens* shaded according to sequence conservation. The sequences were aligned with Muscle (Edgar, 2004) with manual adjustments based on the respective structures. The secondary structure is shown for CodB. Residues interacting with the substrate are denoted by triangles (▼ for CodB and ▲ for Mhp1), residues interacting with the sodium ion through the carbonyl oxygen are shown as ★ and those interacting through the side chain are denoted by ●.

 © 2022 The Authors

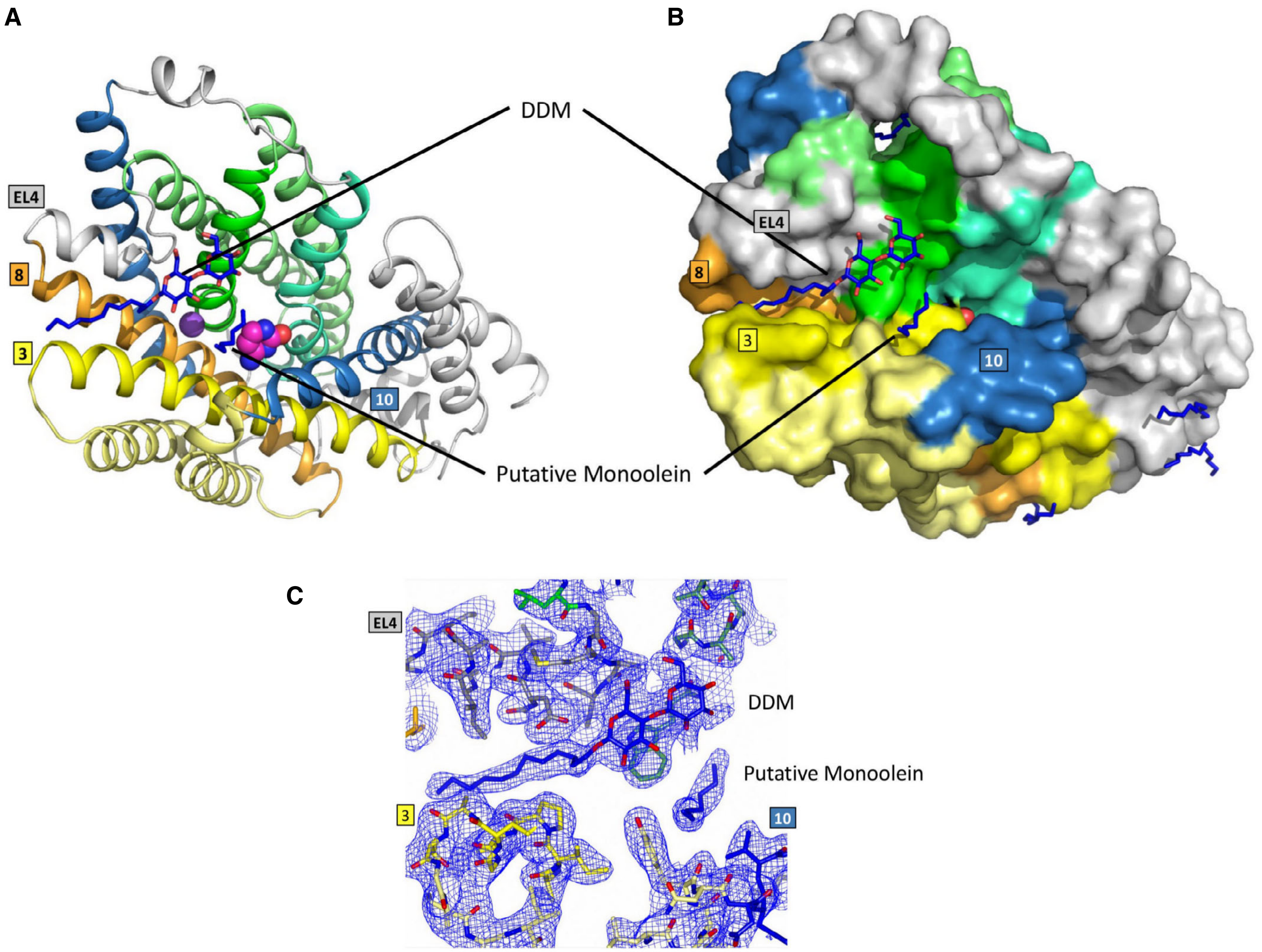

Figure EV2. Detergent and lipid binding in CodB.

As Fig 2B but showing positions of monoolein and DDM that have been putatively modelled in the binding site. Lipids are shown with blue carbon atoms.

A–C (A) cartoon representation, (B) surface representation. (C) Electron density for lipid-like molecules in the cavity. The 2mFo-DFc map is based on phases from the refined structure and contoured at 0.75σ.

© 2022 The Authors

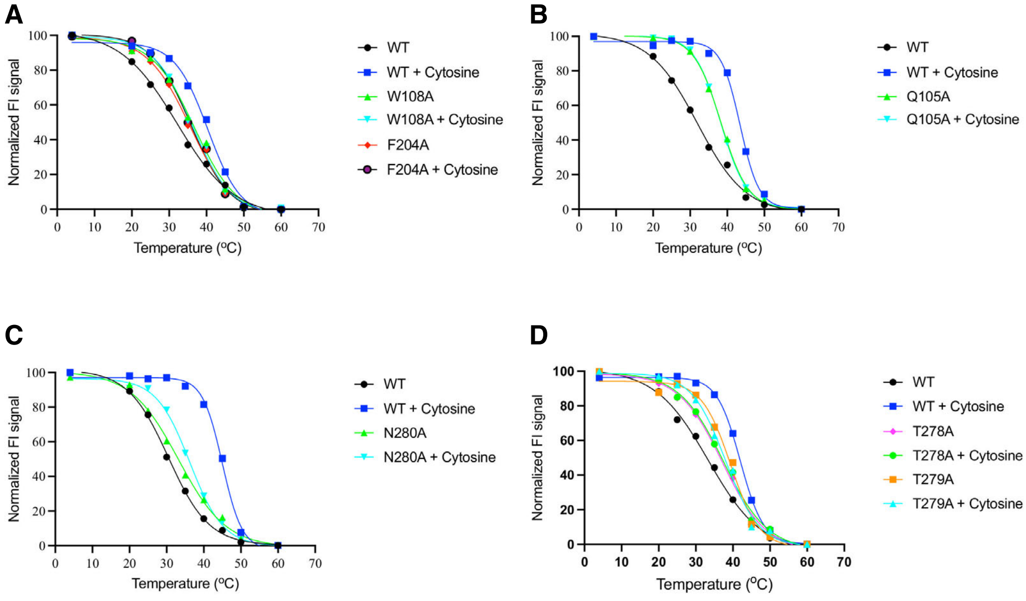

E

	Difference in melting temperature (°C)
WT	10
W108A	-1
F204A	0
Q105A	0
N280A	3
T278A	1
T279A	-1

Figure EV3. Characterisation of CodB mutants using the thermostability assay.

A–D Melting curves for wild-type and mutant proteins with and without cytosine bound using the GFP-TS assay. Values plotted are the averages from $n = 2$ independent titrations, each with two technical repeats.

E Table summarising the data from the graphs in (A–D) showing differences in melting temperatures between each protein with and without cytosine bound.

 © 2022 The Authors

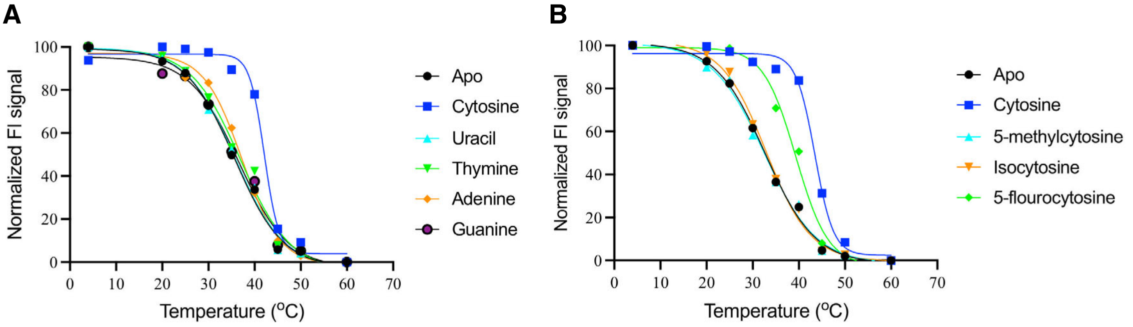

C

Compound	Difference in melting temperature (°C)
1 mM Cytosine	+11
1 mM Uracil	+0
1 mM Thymine	+1
1 mM Adenine	+2
1 mM Guanine	+1
1 mM 5-methylytosine	+0
1 mM Isocytosine	+0
1 mM 5-fluorocytosine	+7

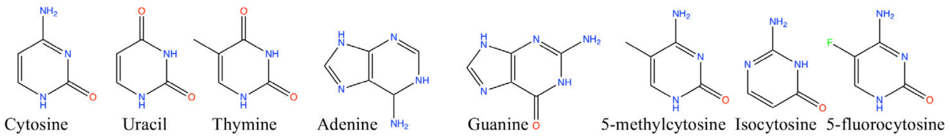

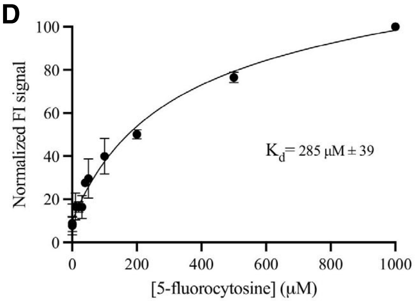

Figure EV4. Characterisation of nucleobase binding to CodB using the thermostability assay.

A, B Melting curves for the wild-type CodB in the presence and absence of selected nucleobases using the GFP-TS assay. Values plotted are the averages from n = 2 independent titrations, each with two technical repeats.

C Table summarising the graphs from (A, B) showing differences in melting temperatures upon addition of the specified nucleobase.

D Binding affinity of CodB for 5-fluorocytosine as measured using the thermostability assay. The K_d was estimated to be 285 ± 39 μM. The measurements are the average of 3 independent titrations with error bars of the s.e.m.

Figure EV5. Sequence conservation in CodB.

A A BLAST search was carried out against UniProt clusters (UniRef90). The most diverse sequences from the 200 sequences deriving from that search (see methods) are shown. Residues that are identical in all 10 sequences are highlighted colored according to ClustalX. Residues interacting with the ligand are shown by a solid triangle (▼), those interacting with the sodium ion through their side chains are shown as a ● and those involved in hydrogen bonds between the helices of the bundle and hash domain respectively are denoted with a ✶. As more diverse sequences are added R216 and Y285 remain constant while there is more divergence for other residues.

B Conservation plotted on the structure of CodB. Residues that are identical in (A) have been coloured blue with N275, N282, R216 and Y285 depicted as red sticks.

C Interaction between Arg216 and Tyr285 coloured as in Fig 2. Hydrogen bonds are shown as dashed lines.

▶

© 2022 The Authors

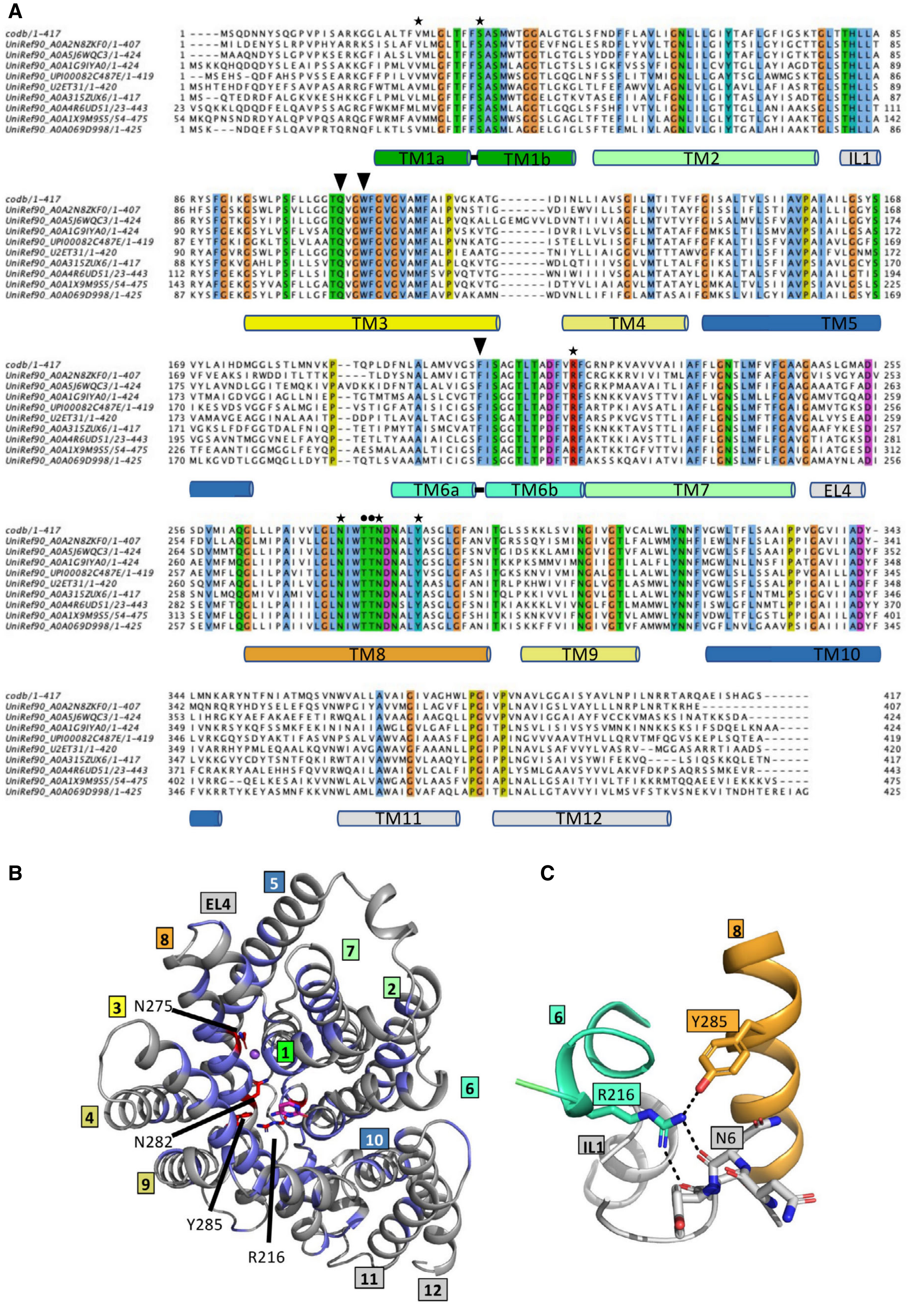

Figure EV5.

 © 2022 The Authors
