# Supplementary material for: Structure of cytosine transport protein CodB provides insight into nucleobase‐cation symporter 1 mechanism
Source: EMBO J. 2022 Jul 1;41(16):e110527. doi: 10.15252/embj.2021110527 (PMC9379551; doi:10.15252/embj.2021110527)
Supplement: Supplementary file 3 [file EMBJ-41-e110527-s002.pdf]

THE EMBO JOURNAL

Structure of cytosine transport protein CodB provides insight into nucleobase-cation symporter 1 mechanism

Caitlin Hatton, Deborah Brotherton, Mahalah Spencer, and Alexander Cameron
OI: 10.15252/embj.2021110527

Corresponding author: Alexander Cameron (a.cameron@warwick.ac.uk)

Review Timeline:

Submission Date:	23rd Dec 21
Editorial Decision:	8th Feb 22
Revision Received:	1st May 22
Accepted:	26th May 22

Editor: Kelly Anderson

Transaction Report:

(Note: With the exception of the correction of typographical or spelling errors that could be a source of ambiguity, letters and reports are not edited. Depending on transfer agreements, referee reports obtained elsewhere may or may not be included in this compilation. Referee reports are anonymous unless the Referee chooses to sign their reports.)

1st Editorial Decision **8th Feb 2022**

Dear Prof. Cameron,

Thank you for submitting your manuscript for consideration by the EMBO Journal. It has now been seen by three referees whose comments are shown below.

I'm happy to invite you to submit a revised version of the manuscript, addressing the comments of all three reviewers. While overall the reviewer comments are positive, some more experiments are needed to bring the manuscript to the level of final publication in the EMBO Journal. It would be good to discuss your plan to address the comments and I am happy to do so with you either by email or zoom.

When preparing your letter of response to the referees' comments, please bear in mind that this will form part of the Review Process File, and will therefore be available online to the community. For more details on our Transparent Editorial Process, please visit our website: https://www.embopress.org/page/journal/14602075/authorguide#transparentprocess

I have attached a guide for revisions for your convenience.

We generally allow three months as standard revision time. As a matter of policy, competing manuscripts published during this period will not negatively impact on our assessment of the conceptual advance presented by your study. However, we request that you contact the editor as soon as possible upon publication of any related work, to discuss how to proceed. Should you foresee a problem in meeting this three-month deadline, please let us know in advance and we may be able to grant an extension.

Thank you for the opportunity to consider your work for publication. I look forward to your revision.

Kind regards,

Kelly

Kelly M Anderson, PhD
Editor
The EMBO Journal
k.anderson@embojournal.org

Further information is available in our Guide For Authors: https://www.embopress.org/page/journal/14602075/authorguide

We realize that it is difficult to revise to a specific deadline. In the interest of protecting the conceptual advance provided by the work, we recommend a revision within 3 months (9th May 2022). Please discuss the revision progress ahead of this time with the editor if you require more time to complete the revisions. Use the link below to submit your revision:

https://emboj.msubmit.net/cgi-bin/main.plex

Referee #1:

This is a clearly written manuscript describing structural characterisation of the CodB cytosine transporter from Proteus vulgaris, as a representative of these proteins that are present in many bacterial species. CodB is only the second member (after Mhp1) of the NCS1 transported family for which the structure has been determined and the authors provide an informative contrast of the structure with that of Mhp1, broadening the signficance of the research. The structure determination and analysis is nicely complemented by transport and stabilisation (binding) assays with wild-type and mutaant proteins, and with a range of substrate analogues. The authors' conclusions are internally consistent and are well supported by their data. My only concerns are minor:

1. The second paragraph of the introduction, referring to the different domains and transmembrane helices of Mhp1 as well as to the structural changes likely to occur during transport, would be easier to follow with a figure. Otherwise, the details in this paragraph are not really needed for the reader to follow the results, and the text could be simplified.
2. The abstract and introduction refers to the transport of 5-fluorouracil via CodB as a potential antibacterial tool. The authors could consider returning to this in the Discussion - how does their work affect the potential use of 5-fluoruracil? In this context it would also be helpful to comment on the degree of similarity between the solved P vulgaris CodB structure and CodB from pathogenic bacteria ie. their model is valid for other CodBs. (Fig. S5 indicates that it is likely to be well conserved.)

3. In the last sentence of the section, "Molecular recognition of CodB" do the authors mean, "No binding was observed for purine bases"? - as cytosine and fluorocytosine bind. More importantly in this section, the Kd for fluorocytosine is high (285 uM) does that have any relevance to point 2 above?
4. The discussion in some places, such as the role of the sodium ion, seemed rather inconclusive and involved quite extensive consideration of other transporters. The authors could consider shortening this.
5. In the first paragraph of the introduction, stating that CodB is found in an operon with CodA, it should be made clear that this is in E coli (for the cited reference) as the operonic arrangement may not be the case in all species. Likewise in Figure 1, regulation by NAC may not be the case in all species.

Referee #2:

The goal of the project described here was to determine how cytosine binds to the CodB transporter from Proteus vulgaris. This is interesting because the substrate analogue 5-fluorocytosine is a novel drug which enters the pathogen Pseudomonas aeruginosa via its homologous transporter. The new structural information could potentially serve to design more potent drugs.

The crystal structure of the cytosine-bound Proteus vulgaris transporter was determined. This enabled the identification of the molecular determinants of cytosine binding of the transporter. The conclusions are supported by the data presented.

It was shown that Proteus vulgaris transporter CodB carries out the sodium-dependent uptake of cytosine and can bind 5-fluorocytosine. Comparison of the substrate-bound structures of CodB and the hydantoin transporter Mhp1, the only other NCS1 family member for which the structure is known, highlight the importance of the hydrogen bonds that the substrates make with the main chain at the breakpoint in the discontinuous helix, TM6. In contrast to other LeuT superfamily members, neither CodB nor Mhp1 make specific interactions with residues on TM1. These results indicate that even though the overall transport mechanism by members of the NCS1 family is similar to the NSS transporters belonging to the same superfamily, the details of the coupling of the substrate flux to that of sodium differ. The new structural information could potentially serve to design more potent drugs targeting the pathogen Pseudomonas aeruginosa. Even though the results confirm insights previously obtained with Mhp1, the data useful to illustrate that the various NCS1 members have a similar transport mechanism.

Concerns
The crystal structure shows the cytosine substrate sandwiched between two aromatic amino acid residues located on TM3 and TM6, respectively. This suggest that the pi-stacking arrangement of the nucleobase between the two aromatic residues is important. Functional studies on mutants of these two residues showed that the impact of the mutation of Phe204 of TM6 to Ala on transport was much less drastic compared to the similar mutation of Trp108 of TM3 (Fig. 6a). A similar observation was made with an equivalent mutation in Mhp1 (Simmons et al., 2014). The Authors suggest that, while important for shaping the pocket, the prime binding site on the bundle domain involves the main chain atoms of TM6 and the side-chain of Trp108. However, an alternative explanation is that upon mutation of Phe204 to Ala a structural change occurs so that another liganding group can take the place of Phe204. Ideally this could be tested by determining the crystal structure of the W204A mutant and this alternative explanation should at least be discussed.
In the legend to Fig. 1, the Authors claim that transport is driven by an inward sodium gradient. What they show is that external sodium is required for the transport process. To substantiate that it is the inward sodium gradient which drives the transport it is necessary to do more experiments. The simplest one is to show that an ionophore such as nigericin, expected to collapse the sodium gradient, inhibits transport.
The density at the site equivalent to the Na2 site of other members of the LeuT superfamily, suggest that this indeed represents the sodium binding site of CodB. It is important to substantiate this by testing the functional impact of mutation of Thr 278 and Thr279 from TM8 on transport.
Coupling of sodium and substrate fluxes appear to be different between NSS transporters and those of the NCS family. Both appear to have the same Na2 site and sodium binding to this site render the transporters outward-facing because this binding promotes the interaction between TMs 1 and 8. In the NSS transporters the subsequent addition of substrate overcomes this interaction rendering the transporter inward-facing capable of releasing the substrate on the inside. Since substrate binding by the NCS1 transporters does not involve TM1 there must be a different mechanism by which the substrate causes the transporter to become inward-facing. The Authors should at least speculate on how this could be happening.

Minor concerns
Is radio-labelled 5-fluoro cytosine commercially available? This would enable to show that this compound not only binds but in fact is transported, as expected.
In the beginning of the Results section the is some confusion, for instance with the comparisons of the diverse panels of Fig. 1 between the text and the legend.

Referee #3:

The authors report a crystal structure of a cytosine transporter CodB from Proteus vulgaris in complex with cytosine, together

with transport assays and site-directed mutagenesis. These results provide detailed insights into molecular recognition and transport in CodB. By extension, this work increases understanding of substrate recognition and transport in the NCS1 family of transporters. Overall, this is an interesting study based on solid data. The following points need to be addressed before publication.

Crystallography data in Table 1. The datasets have unusually high Rmerge values (0.5 and 0.3739). Could it be possible that the space group was incorrectly assigned? How about the statistics of data processing with different space groups (with reduced symmetry)? The high-resolution shell had very low signal-to-noise ratio (0.9/0.98) and very high Rmerge. The resolutions were thus over estimated. The authors should truncate the reflections further to lower resolutions with more acceptable statistics.

It is important to show refined electron density for the entire protein as a supplementary figure.

The crystal structure nicely revealed the sodium-binding site. It would be important to validate critical residues (such as Thr278) constituting the sodium-binding site by mutagenesis and cytosine binding and transport assays.

Labels in Fig. 1 (Fig.1 a-c) are inconsistent.

In Fig. 3, label 3a is missing.

We thank all the referees for their positive and helpful comments. We have addressed the concerns as described below. The amendments to the text based on the referees comments are shown with tracked changes.

Referee #1:

This is a clearly written manuscript describing structural characterisation of the CodB cytosine transporter from Proteus vulgaris, as a representative of these proteins that are present in many bacterial species. CodB is only the second member (after Mhp1) of the NCS1 transported family for which the structure has been determined and the authors provide an informative contrast of the structure with that of Mhp1, broadening the signficance of the research. The structure determination and analysis is nicely complemented by transport and stabilisation (binding) assays with wild-type and mutaant proteins, and with a range of substrate analogues. The authors' conclusions are internally consistent and are well supported by their data. My only concerns are minor:

1. The second paragraph of the introduction, referring to the different domains and transmembrane helices of Mhp1 as well as to the structural changes likely to occur during transport, would be easier to follow with a figure. Otherwise, the details in this paragraph are not really needed for the reader to follow the results, and the text could be simplified.

We thank the referee for this useful comment. Rather than include a figure, we have simplified the text as the more complicated description of the protein is not really required here, as pointed out by the Referee, and is discussed elsewhere in the text associated with the relevant figures.

2. The abstract and introduction refers to the transport of 5-fluorouracil via CodB as a potential antibacterial tool. The authors could consider returning to this in the Discussion - how does their work affect the potential use of 5-fluoruracil? In this context it would also be helpful to comment on the degree of similarity between the solved P vulgaris CodB structure and CodB from pathogenic bacteria ie. their model is valid for other CodBs. (Fig. S5 indicates that it is likely to be well conserved.)

We have amended the discussion slightly to include this, pointing out the similarity between the two sequences.

3. In the last sentence of the section, "Molecular recognition of CodB" do the authors mean, "No binding was observed for purine bases"? - as cytosine and fluorocytosine bind. More importantly in this section, the Kd for fluorocytosine is high (285 uM) does that have any relevance to point 2 above?

Thanks for spotting this - it should read purines. Given the similarity between CodB from *P aeruginosa* and *P vulgaris* we would be surprised if there would be a big difference in the affinity for 5-fluorocytosine between these two organisms. Clearly the 5-fluorocytosine is able to be transported by CodB from *P aeruginosa*.

4. The discussion in some places, such as the role of the sodium ion, seemed rather inconclusive and involved quite extensive consideration of other transporters. The authors could consider shortening this.

Given this comment, we have tried to streamline the text in line with the referee's comments.

5. In the first paragraph of the introduction, stating that CodB is found in an operon with CodA, it should be made clear that this is in E coli (for the cited reference) as the operonic arrangement may not be the case in all species. Likewise in Figure 1, regulation by NAC may not be the case in all species.

This is a good point. As for *E coli,* sequence analysis shows that CodB from both *P. vulgaris* and *P. aeruginosa* is found together with CodA. We have modified the text to specifically refer to *E coli* in the

discussion of NAC.

Referee #2:

The goal of the project described here was to determine how cytosine binds to the CodB transporter from Proteus vulgaris. This is interesting because the substrate analogue 5-fluorocytosine is a novel drug which enters the pathogen Pseudomonas aeruginosa via its homologous transporter. The new structural information could potentially serve to design more potent drugs.

The crystal structure of the cytosine-bound Proteus vulgaris transporter was determined. This enabled the identification of the molecular determinants of cytosine binding of the transporter. The conclusions are supported by the data presented.

It was shown that Proteus vulgaris transporter CodB carries out the sodium-dependent uptake of cytosine and can bind 5-fluorocytosine. Comparison of the substrate-bound structures of CodB and the hydantoin transporter Mhp1, the only other NCS1 family member for which the structure is known, highlight the importance of the hydrogen bonds that the substrates make with the main chain at the breakpoint in the discontinuous helix, TM6. In contrast to other LeuT superfamily members, neither CodB nor Mhp1 make specific interactions with residues on TM1. These results indicate that even though the overall transport mechanism by members of the NCS1 family is similar to the NSS transporters belonging to the same superfamily, the details of the coupling of the substrate flux to that of sodium differ. The new structural information could potentially serve to design more potent drugs targeting the pathogen Pseudomonas aeruginosa. Even though the results confirm insights previously obtained with Mhp1, the data useful to illustrate that the various NCS1 members have a similar transport mechanism.

Concerns
The crystal structure shows the cytosine substrate sandwiched between two aromatic amino acid residues located on TM3 and TM6, respectively. This suggest that the pi-stacking arrangement of the nucleobase between the two aromatic residues is important. Functional studies on mutants of these two residues showed that the impact of the mutation of Phe204 of TM6 to Ala on transport was much less drastic compared to the similar mutation of Trp108 of TM3 (Fig. 6a). A similar observation was made with an equivalent mutation in Mhp1 (Simmons et al., 2014). The Authors suggest that, while important for shaping the pocket, the prime binding site on the bundle domain involves the main chain atoms of TM6 and the side-chain of Trp108. However, an alternative explanation is that upon mutation of Phe204 to Ala a structural change occurs so that another liganding group can take the place of Phe204. Ideally this could be tested by determining the crystal structure of the W204A mutant and this alternative explanation should at least be discussed.

This is a fair point and we agree with the referee. We now discuss this possibility in the text.

In the legend to Fig. 1, the Authors claim that transport is driven by an inward sodium gradient. What they show is that external sodium is required for the transport process. To substantiate that it is the inward sodium gradient which drives the transport it is necessary to do more experiments. The simplest one is to show that an ionophore such as nigericin, expected to collapse the sodium gradient, inhibits transport.

Following the referee's suggestion we included the sodium-specific ionophore monensin as a control in the assay. This showed drastically reduced uptake of the ligand. We have included this information as a supplementary figure.

The density at the site equivalent to the Na2 site of other members of the LeuT superfamily, suggest that

this indeed represents the sodium binding site of CodB. It is important to substantiate this by testing the functional impact of mutation of Thr 278 and Thr279 from TM8 on transport.

We have now carried out the transport assay using individual mutations of Thr278 and Thr279 as well as a double mutant (Fig 6). Mutating either Thr279 or both to alanine drastically reduces transport. Under the conditions of the assay, however, mutation of Thr278 has little effect on the transport. We also now show that neither of the single mutations is stabilised by the binding of cytosine in the stability assay in contrast to the wild type protein (Fig EV3D).

Coupling of sodium and substrate fluxes appear to be different between NSS transporters and those of the NCS family. Both appear to have the same Na2 site and sodium binding to this site render the transporters outward-facing because this binding promotes the interaction between TMs 1 and 8. In the NSS transporters the subsequent addition of substrate overcomes this interaction rendering the transporter inward-facing capable of releasing the substrate on the inside. Since substrate binding by the NCS1 transporters does not involve TM1 there must be a different mechanism by which the substrate causes the transporter to become inward-facing. The Authors should at least speculate on how this could be happening.

The text has now been modified to clarify this point. The switch from inward to outward-facing and *vice versa* is caused by the movement of the whole of the hash motif (TMs 3,4,8,9) relative to the bundle (TMS 1,2,6,7). Upon substrate binding TM10 will move, pulling on TM9 of the hash domain. It can be speculated that this will affect the position of the hash domain. There is no necessity for TM1 to be involved as the substrate still spans TM6 of the bundle domain and TM3 of the hash domain.

Minor concerns

Is radio-labelled 5-fluoro cytosine commercially available? This would enable to show that this compound not only binds but in fact is transported, as expected.

Unfortunately, we have not been able to obtain radio-labelled 5-fluorocytosine.

In the beginning of the Results section the is some confusion, for instance with the comparisons of the diverse panels of Fig. 1 between the text and the legend.

Thanks for pointing this out. This has been modified.

Referee #3:

The authors report a crystal structure of a cytosine transporter CodB from Proteus vulgaris in complex with cytosine, together with transport assays and site-directed mutagenesis. These results provide detailed insights into molecular recognition and transport in CodB. By extension, this work increases understanding of substrate recognition and transport in the NCS1 family of transporters. Overall, this is an interesting study based on solid data. The following points need to be addressed before publication.

Crystallography data in Table 1. The datasets have unusually high Rmerge values (0.5 and 0.3739). Could it be possible that the space group was incorrectly assigned? How about the statistics of data processing with different space groups (with reduced symmetry)? The high-resolution shell had very low signal-to-noise ratio (0.9/0.98) and very high Rmerge. The resolutions were thus over estimated. The authors should truncate the reflections further to lower resolutions with more acceptable statistics.

We thank the referee for drawing our attention to this. In collecting the data we collected many more images than were actually used in the final data as we observed the resolution limit of the crystal

reduced over time. We therefore reduced the number of images used in the final refined data set. However, in creating Table 1 (using the Phenix software) we inadvertently uploaded a file containing all images. Consequently, the Rmerge values are very high. However, we prefer to use the CC0.5 statistic in evaluating the usefulness of the data as described in this article Curr Opin Struct Biol. 2015 Oct; 34: 60–68. Even in the data we include in the original table the CC0.5 is a respectable value (the suggested cut-off in Aimless, for example is where this drops to below 0.3), as is Rpim. In the data we used during the refinement (as amended in the current table) all the statistics are within acceptable limits. There is no evidence of twinning in any of the statistical tests output by the processing programs and the R-factor and R-free are reasonable for this resolution data.

The most important criteria however is the quality of the density. We omitted the cytosine ligand and refined the structure against data extending to 2.4Å, 2.5Å and 2.6Å and evaluated the maps. While the difference is marginal, the quality of the data is generally better for the 2.4Å data set (see below).

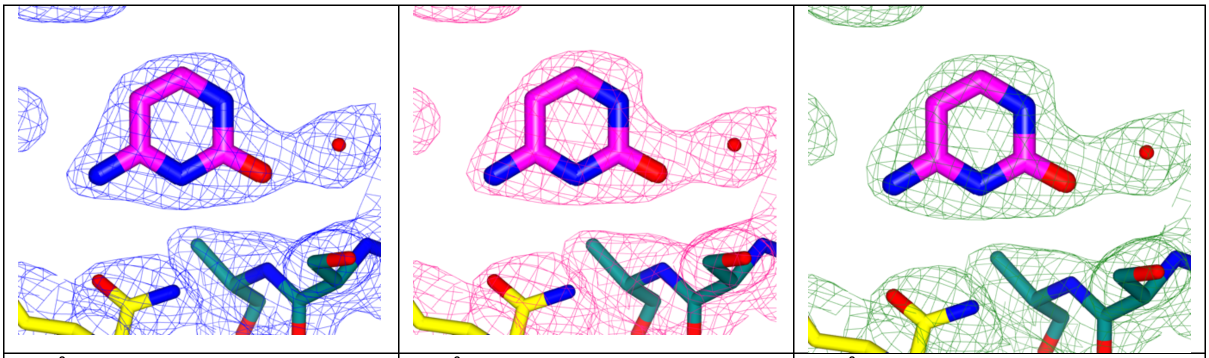

2.4Å: note how the density follows the cytosine	2.5Å	2.6Å

It is important to show refined electron density for the entire protein as a supplementary figure.

We have now included this as a supplementary figure.

The crystal structure nicely revealed the sodium-binding site. It would be important to validate critical residues (such as Thr278) constituting the sodium-binding site by mutagenesis and cytosine binding and transport assays.

These have now been included as described above.

Labels in Fig. 1 (Fig.1 a-c) are inconsistent.

Altered, thank you.

In Fig. 3, label 3a is missing.

Thanks for pointing this out. This has now been added.

1st Revision - Editorial Decision 26th May 2022

Dear Alex,

Congratulations on an excellent manuscript, I am pleased to inform you that your manuscript has been accepted for publication in The EMBO Journal. Thank you for your comprehensive response to the referee concerns. You will notice one referee remains concerned regarding the overall Rmerge value. We therefore sought advice from an additional expert in protein crystallography who suggested these are typical data sets, especially considering your CC-1/2 was in acceptable resolution range and Rfree was not higher. This advisor suggested it might help to include the Wilson plot as a supplementary figure and we agree this would be useful data to include in the final version.

We include a synopsis of the paper online (see http://emboj.embopress.org/). For this, please provide me with a general summary statement and 3-5 bullet points that capture the key findings of the paper. We also need a summary figure for the synopsis. The size should be 550 wide by 200-440 high (pixels). You can also use something from the figures if that is easier.

I will begin the final checks on your manuscript before submitting to the publisher next week. Once at the publisher, it will take about 3 weeks for your manuscript to be published online. As a reminder, the entire review process including referee concerns and your point-by-point response will be available to readers.

It has been a pleasure to work with you to get this to the acceptance stage. I will be in touch throughout the final editorial process until publication. In the meantime, I hope you find time to celebrate!

Kind regards,

Kelly

Kelly M Anderson, PhD
Editor
The EMBO Journal
k.anderson@embojournal.org

Referee #3:

The manuscript is improved. Again, very interesting work.

However, my concern about the crystallography data in Table 1 remains.

The authors reduced the number of images (reflections) to improve slightly the Rmerge values (from 0.5 and 0.3739) to (0.5/0.1829). However, the 'multiplicity' of the reflections is only 5.2 or 3.5. In other words, the redundancy of measurements of symmetry related reflections is not high but the Rmerge values are alarmingly high. An overall Rmerge value of 0.5 strongly indicates issues such as incorrect space group assignment. The electron density does look ok.

Please note that it is EMBO Journal policy for the transcript of the editorial process (containing referee reports and your response letter) to be published as an online supplement to each paper. If you do NOT want this, you will need to inform the Editorial Office via email immediately. More information is available here: https://www.embopress.org/page/journal/14602075/authorguide#transparentprocess

Your manuscript will be processed for publication in the journal by EMBO Press. Manuscripts in the PDF and electronic editions of The EMBO Journal will be copy edited, and you will be provided with page proofs prior to publication. Please note that supplementary information is not included in the proofs.

Please note that you will be contacted by Wiley Author Services to complete licensing and payment information. The required 'Page Charges Authorization Form' is available here: https://www.embopress.org/pb-assets/embo-site/tej_apc.pdf

Should you be planning a Press Release on your article, please get in contact with embojournal@wiley.com as early as possible, in order to coordinate publication and release dates.

If you have any questions, please do not hesitate to call or email the Editorial Office. Thank you for your contribution to The EMBO Journal.

** Click here to be directed to your login page: https://emboj.msubmit.net

Response to Referee

Response in blue

The manuscript is improved. Again, very interesting work.

However, my concern about the crystallography data in Table 1 remains.

The authors reduced the number of images (reflections) to improve slightly the Rmerge values (from 0.5 and 0.3739) to (0.5/0.1829). However, the 'multiplicity' of the reflections is only 5.2 or 3.5. In other words, the redundancy of measurements of symmetry related reflections is not high but the Rmerge values are alarmingly high. An overall Rmerge value of 0.5 strongly indicates issues such as incorrect space group assignment. The electron density does look ok.

We have looked closely at the data. It is noticeable that the Rpim, which takes account of the multiplicity of reflections is actually lower than the Rmerge. Our experience with crystals grown using LCP and collected on the microfocus beamline tend to have high Rmerge values even when merged as P1. The various plots and analysis that are output by ctruncate do not indicate twinning. Below we show the Wilson Plot and L-test for twinning as output from the CCP4i2 interface, which show no evidence of twinning. Importantly also, the Rfree value of the final refined structure is consistent with that expected for the resolution of the data and would be expected to be much higher were the data to be twinned.

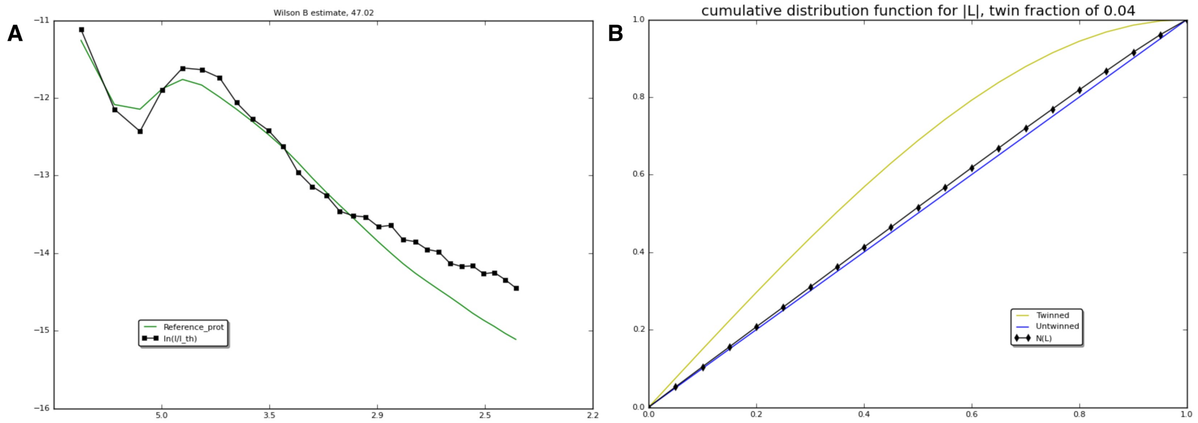

Dear Alex,

Thank you for providing the final response to reviewers and including the recommended graph. We feel this new data adequately addresses the referee's concern and are therefore happy to move forward with publication of this manuscript.

I will be in touch if there are any remaining issues.

All the best,
Kelly

EMBO Press Author Checklist

Corresponding Author Name: Alexander Cameron	
Journal Submitted to: EMBO	
Manuscript Number: EMBOJ-2021-110527R	

USEFUL LINKS FOR COMPLETING THIS FORM
The EMBO Journal - Author Guidelines
EMBO Reports - Author Guidelines
Molecular Systems Biology - Author Guidelines
EMBO Molecular Medicine - Author Guidelines

Reporting Checklist for Life Science Articles (updated January 2022)
This checklist is adapted from Materials Design Analysis Reporting (MDAR) Checklist for Authors. MDAR establishes a minimum set of requirements in transparent reporting in the life sciences (see Statement of Task: 10.31222/osf.io/9sm4x). Please follow the journal's guidelines in preparing your manuscript.
Please note that a copy of this checklist will be published alongside your article.

Abridged guidelines for figures
1. Data
The data shown in figures should satisfy the following conditions:
- the data were obtained and processed according to the field's best practice and are presented to reflect the results of the experiments in an accurate and unbiased manner.
- ideally, figure panels should include only measurements that are directly comparable to each other and obtained with the same assay.
- plots include clearly labeled error bars for independent experiments and sample sizes. Unless justified, error bars should not be shown for technical replicates.
- if n<5, the individual data points from each experiment should be plotted. Any statistical test employed should be justified.
- Source Data should be included to report the data underlying figures according to the guidelines set out in the authorship guidelines on Data Presentation.

2. Captions
Each figure caption should contain the following information, for each panel where they are relevant:
- a specification of the experimental system investigated (eg cell line, species name).
- the assay(s) and method(s) used to carry out the reported observations and measurements.
- an explicit mention of the biological and chemical entity(ies) that are being measured.
- an explicit mention of the biological and chemical entity(ies) that are altered/varied/perturbed in a controlled manner.
- the exact sample size (n) for each experimental group/condition, given as a number, not a range;
- a description of the sample collection allowing the reader to understand whether the samples represent technical or biological replicates (including how many animals, litters, cultures, etc.).
- a statement of how many times the experiment shown was independently replicated in the laboratory.
- definitions of statistical methods and measures:
  - common tests, such as t-test (please specify whether paired vs. unpaired), simple χ2 tests, Wilcoxon and Mann-Whitney tests, can be unambiguously identified by name only, but more complex techniques should be described in the methods section;
  - are tests one-sided or two-sided?
  - are there adjustments for multiple comparisons?
  - exact statistical test results, e.g., P values = x but not P values < x;
  - definition of 'center values' as median or average;
  - definition of error bars as s.d. or s.e.m.

Please complete ALL of the questions below.
Select "Not Applicable" only when the requested information is not relevant for your study.

Materials

Newly Created Materials	Information included in the manuscript?	In which section is the information available? (Reagents and Tools Table, Materials and Methods, Figures, Data Availability Section)
New materials and reagents need to be available; do any restrictions apply?	Not Applicable	

Antibodies	Information included in the manuscript?	In which section is the information available? (Reagents and Tools Table, Materials and Methods, Figures, Data Availability Section)
For **antibodies** provide the following information:<br>- Commercial antibodies: RRID (if possible) or supplier name, catalogue number and or/clone number<br>- Non-commercial: RRID or citation	Not Applicable	

DNA and RNA sequences	Information included in the manuscript?	In which section is the information available? (Reagents and Tools Table, Materials and Methods, Figures, Data Availability Section)
**Short novel DNA or RNA including primers, probes**: provide the sequences.	Yes	Appendix

Cell materials	Information included in the manuscript?	In which section is the information available? (Reagents and Tools Table, Materials and Methods, Figures, Data Availability Section)
**Cell lines:** Provide species information, strain. Provide accession number in repository **OR** supplier name, catalog number, clone number, and/**OR** RRID.	Yes	Materials and Methods
**Primary cultures:** Provide species, strain, sex of origin, genetic modification status.	Not Applicable	
Report if the cell lines were recently **authenticated** (e.g., by STR profiling) and tested for mycoplasma contamination.	Not Applicable	

Experimental animals	Information included in the manuscript?	In which section is the information available? (Reagents and Tools Table, Materials and Methods, Figures, Data Availability Section)
**Laboratory animals or Model organisms:** Provide species, strain, sex, age, genetic modification status. Provide accession number in repository **OR** supplier name, catalog number, clone number, **OR** RRID.	Not Applicable	
**Animal observed in or captured from the field:** Provide species, sex, and age where possible.	Not Applicable	
Please detail **housing and husbandry conditions**.	Not Applicable	

Plants and microbes	Information included in the manuscript?	In which section is the information available? (Reagents and Tools Table, Materials and Methods, Figures, Data Availability Section)
**Plants:** provide species and strain, ecotype and cultivar where relevant, unique accession number if available, and source (including location for collected wild specimens).	Not Applicable	
**Microbes:** provide species and strain, unique accession number if available, and source.	Yes	Materials and Methods

Human research participants	Information included in the manuscript?	In which section is the information available? (Reagents and Tools Table, Materials and Methods, Figures, Data Availability Section)
If collected and within the bounds of privacy constraints report on age, sex and gender or ethnicity for all study participants.	Not Applicable	

Core facilities	Information included in the manuscript?	In which section is the information available? (Reagents and Tools Table, Materials and Methods, Figures, Data Availability Section)
If your work benefited from core facilities, was their service mentioned in the acknowledgments section?	Yes	Materials and Methods, Acknowledgements

Design

Study protocol	Information included in the manuscript?	In which section is the information available? (Reagents and Tools Table, Materials and Methods, Figures, Data Availability Section)
If study protocol has been **pre-registered, provide DOI in the manuscript**. For clinical trials, provide the trial registration number **OR** cite DOI.	Not Applicable	
Report the **clinical trial registration number** (at ClinicalTrials.gov or equivalent), where applicable.	Not Applicable	

Laboratory protocol	Information included in the manuscript?	In which section is the information available? (Reagents and Tools Table, Materials and Methods, Figures, Data Availability Section)
Provide DOI OR other citation details if **external detailed step-by-step protocols** are available.	Not Applicable	

Experimental study design and statistics	Information included in the manuscript?	In which section is the information available? (Reagents and Tools Table, Materials and Methods, Figures, Data Availability Section)
Include a statement about **sample size** estimate even if no statistical methods were used.	Yes	Materials and Methods / Figure Legends
Were any steps taken to minimize the effects of subjective bias when allocating animals/samples to treatment (e.g. **randomization procedure**)? If yes, have they been described?	Not Applicable	
Include a statement about **blinding** even if no blinding was done.	Not Applicable	
Describe **inclusion/exclusion criteria** if samples or animals were excluded from the analysis. Were the criteria pre-established?<br><br>If sample or data points were omitted from analysis, report if this was due to attrition or intentional exclusion and provide justification.	Not Applicable	
For every figure, are **statistical tests** justified as appropriate? Do the data meet the assumptions of the tests (e.g., normal distribution)? Describe any methods used to assess it. Is there an estimate of variation within each group of data? Is the variance similar between the groups that are being statistically compared?	Not Applicable	

Sample definition and in-laboratory replication	Information included in the manuscript?	In which section is the information available? (Reagents and Tools Table, Materials and Methods, Figures, Data Availability Section)
In the figure legends: state number of times the experiment was **replicated** in laboratory.	Yes	Materials and Methods / Figure Legends
In the figure legends: define whether data describe **technical or biological replicates**.	Yes	Materials and Methods / Figure Legends

Ethics

Ethics	Information included in the manuscript?	In which section is the information available? (Reagents and Tools Table, Materials and Methods, Figures, Data Availability Section)
Studies involving **human participants**: State details of **authority granting ethics approval** (IRB or equivalent committee(s), provide reference number for approval.	Not Applicable	
Studies involving **human participants**: Include a statement confirming that **informed consent** was obtained from all subjects and that the experiments conformed to the principles set out in the WMA Declaration of Helsinki and the Department of Health and Human Services Belmont Report.	Not Applicable	
Studies involving **human participants:** For publication of **patient photos**, include a statement confirming that consent to publish was obtained.	Not Applicable	
Studies involving experimental **animals**: State details of **authority granting ethics approva**l (IRB or equivalent committee(s), provide reference number for approval. Include a statement of compliance with ethical regulations.	Not Applicable	
Studies involving **specimen and field samples:** State if relevant **permits** obtained, provide details of authority approving study; if none were required, explain why.	Not Applicable	

Dual Use Research of Concern (DURC)	Information included in the manuscript?	In which section is the information available? (Reagents and Tools Table, Materials and Methods, Figures, Data Availability Section)
Could your study fall under dual use research restrictions? Please check biosecurity documents and list of **select agents and toxins** (CDC): https://www.selectagents.gov/sat/list.htm	Not Applicable	
If you used a select agent, is the security level of the lab appropriate and reported in the manuscript?	Not Applicable	
If a study is subject to dual use research of concern regulations, is the name of the **authority granting approval and reference number** for the regulatory approval provided in the manuscript?	Not Applicable	

Reporting

The MDAR framework recommends adoption of discipline-specific guidelines, established and endorsed through community initiatives. Journals have their own policy about requiring specific guidelines and recommendations to complement MDAR.

Adherence to community standards	Information included in the manuscript?	In which section is the information available? (Reagents and Tools Table, Materials and Methods, Figures, Data Availability Section)
State if relevant guidelines or checklists (e.g., **ICMJE, MIBBI, ARRIVE, PRISMA**) have been followed or provided.	Not Applicable	
For **tumor marker prognostic studies,** we recommend that you follow the **REMARK** reporting guidelines (see link list at top right). See author guidelines, under 'Reporting Guidelines'. Please confirm you have followed these guidelines.	Not Applicable	
For **phase II and III randomized controlled trials**, please refer to the **CONSORT** flow diagram (see link list at top right) and submit the CONSORT checklist (see link list at top right) with your submission. See author guidelines, under 'Reporting Guidelines'. Please confirm you have submitted this list.	Not Applicable	

Data Availability

Data availability	Information included in the manuscript?	In which section is the information available? (Reagents and Tools Table, Materials and Methods, Figures, Data Availability Section)
Have **primary datasets** been deposited according to the journal's guidelines (see 'Data Deposition' section) and the respective accession numbers provided in the Data Availability Section?	Yes	Data Availability Section
Were **human clinical and genomic datasets** deposited in a public access-controlled repository in accordance to ethical obligations to the patients and to the applicable consent agreement?	Not Applicable	
Are **computational models** that are central and integral to a study available without restrictions in a machine-readable form? Were the relevant accession numbers or links provided?	Not Applicable	
If publicly available data were reused, provide the respective **data citations in the reference list**.	Not Applicable	
